# The impact and cost-effectiveness of combined HIV prevention scenarios among transgender women sex-workers in Lima, Peru: a mathematical modelling study

**DOI:** 10.1016/S2468-2667(18)30236-6

**Published:** 2019-01-23

**Authors:** Annick Bórquez, Juan Vicente Guanira, Paul Revill, Patricia Caballero, Alfonso Silva-Santisteban, Sherrie Kelly, Ximena Salazar, Patricia Bracamonte, Percy Minaya, Timothy B Hallett, Carlos F Cáceres

**Affiliations:** Department of Infectious Disease Epidemiology, Imperial College London, London, UK, Division of Infectious Diseases and Global Public Health, University of California San Diego, San Diego, CA, USA; Investigaciones Médicas en Salud (INMENSA), Lima, Peru; Centre for Health Economics, University of York, York, UK; Instituto Nacional de Salud, Lima, Peru, Ministry of Health, Lima, Peru; Centro de Investigación Interdisciplinaria en Sexualidad, SIDA y Sociedad, Universidad Cayetano Heredia, Lima, Peru; Modelling and Biostatistics, Burnet Institute, Melbourne, VIC, Australia; Centro de Investigación Interdisciplinaria en Sexualidad, SIDA y Sociedad, Universidad Cayetano Heredia, Lima, Peru; UNAIDS, Lima, Peru; Ministry of Health, Lima, Peru; Department of Infectious Disease Epidemiology, Imperial College London, London, UK; Centro de Investigación Interdisciplinaria en Sexualidad, SIDA y Sociedad, Universidad Cayetano Heredia, Lima, Peru

## Abstract

**Background:**

HIV incidence remains high among transgender women in Lima, Peru, most of whom report sex work. On the basis of a stakeholder analysis and health system capacity assessment, we designed a mathematical model to guide HIV programmatic planning among transgender women sex workers (TWSW) in Lima.

**Methods:**

Using a deterministic compartmental model, we modelled HIV transmission among TWSW, their stable partners, and their clients to estimate the impact and cost-effectiveness of combinations of interventions compared with the standard of care on reducing HIV incidence over a 10-year period. We simulated HIV transmission accounting for differences in sexual positioning in anal intercourse and condom use by partner type and fitted the model to HIV surveillance data using Latin hypercube sampling. The interventions we considered were 15% relative increase in condom use with clients and 10% relative increase with stable partners; increase in antiretroviral treatment (ART) coverage at CD4 count lower than 500 cells per mm^3^ and greater than or equal to 500 cells per mm^3^; and 15% pre-exposure prophylaxis (PrEP) coverage using generic and branded formulations. We considered a basic scenario accounting for current limitations in the Peruvian HIV services and an enhanced scenario assuming achievement of the UNAIDS 90–90-90 targets and general improvements in HIV services. The 50 best fits according to log-likelihood were used to give the minimum and maximum values of intervention effect for each combination. We used disability-adjusted life-years (DALYs) to measure the negative health outcomes associated with HIV infection that could be averted through the interventions investigated and calculated incremental cost-effectiveness ratios to compare their cost-effectiveness.

**Findings:**

Under the basic scenario, combining the four interventions of increasing condom use with clients and stable partners, extending ART to people with CD4 count greater than or equal to 500 cells per mm^3^, and 15% PrEP coverage with generic drugs would avert 47% (range 27–51) of new infections in TWSW, their clients, and their stable partners over 10 years, with an incremental cost-effectiveness ratio of US$509 per DALY averted. Under the enhanced scenario, this strategy would avert 61% (44–64) of new infections among this population with an incremental cost-effectiveness ratio of $1003 per DALY averted. Under both scenarios, implementation of this strategy approaches or surpasses the 50% incidence reduction goal and would represent a cost-effective use of country resources if generic PrEP drugs are used. The total cost of implementing this strategy under the enhanced scenario would be approximately $1·2 million per year over 10 years, corresponding to 10% of the current Global Fund’s yearly contribution in Peru.

**Interpretation:**

Investments in HIV services among TWSW in Lima would be cost-effective, even under stringent cost-effectiveness criteria when accounting for setting-specific resource constraints. Notable improvements in HIV testing rates, innovative interventions to increase condom use, and reduced PrEP costs will be key to achieving the 50% incidence reduction goal. Modelling studies incorporating stakeholders’ perspectives and health system assessments can bring added value to HIV policy making.

## Introduction

HIV incidence among men who have sex with men (MSM) and transgender women in Peru remains high at approximately 4%.^1^ Historically, prevention strategies have relied on condom distribution and management of sexually transmitted infections. Although peer-educator outreach proved successful in initially facilitating these interventions, lack of funding and insufficient monitoring and evaluation have diminished their quality and scope. After free antiretroviral treatment (ART) was implemented in Peru in 2004, coverage has increased at a slow pace and remains substandard among key populations, mostly due to very low testing rates and an inefficient process of linkage to care. Despite national guidelines prescribing treatment for individuals with CD4 counts lower than 500 cells per mm^3^ from December, 2014, and extending coverage to include individuals with counts of 500 cells per mm^3^ or more from March, 2018, it was estimated that less than 30% of HIV-positive MSM or transgender women were aware of their HIV status^2^ and of those, less than 80% were on ART.^3–5^

Increased awareness of the shortcomings of the national HIV response has pushed the key population agenda forward, especially focusing on MSM and transgender women. In particular, the need to distinguish transgender women from MSM in programmatic planning has been highlighted because the identity, vulnerabilities to HIV, and prevention needs of those two populations differ. In 2014, the Peruvian Ministry of Health initiated a policy dialogue to introduce combination HIV prevention for key populations, with emphasis on the development of a customised HIV prevention plan for transgender women. In collaboration with the Ministry of Health, UNAIDS, and civil society groups, we implemented a mixed-methods study to support this endeavour within an evidence-based implementation science framework. The study involved a health system capacity evaluation, a stakeholder analysis, and a mathematical modelling analysis to respectively inform on the acceptability, feasibility, and cost-effectiveness of different HIV prevention methods, building on past achievements and incorporating new international guidelines into the current Peruvian context.

The health system capacity evaluation aimed to investigate the status of HIV prevention and delivery of care in Peru in terms of infrastructure, staff capacity, budget allocation, activities, organisation, and outputs. The stakeholder analysis^6,7^ was done to explore perceptions of HIV risk and knowledge of, attitudes towards, and intention to use diverse prevention methods among members of the MSM and transgender women communities, as well as adoption by health professionals and decisions makers. The mathematical modelling study, the focus herein, aimed to provide estimates of impact and cost-effectiveness of the various interventions to identify cost-effective and feasible combinations in the Peruvian setting. Given that most (64%) transgender women in Lima report currently engaging in sex work,^8^ we investigated impact and cost-effectiveness of HIV combination prevention interventions among transgender women sex workers (TWSW) in Lima.

## Methods

### Overview

We identified HIV interventions to be investigated on the basis of the WHO guidelines for HIV prevention among MSM and transgender women and characterised and costed these within the context of TWSW in Lima, taking into account stakeholders’ perspectives and current HIV care system limitations. We modelled the impact and estimated cost-effectiveness of these interventions in isolation and all possible combinations on reducing HIV incidence among TWSW and their partners from 2016 to 2026, compared with a status quo scenario assuming no changes in the availability of HIV services among this population (defined as the standard of care or baseline).

### Model

**Figure 1:**
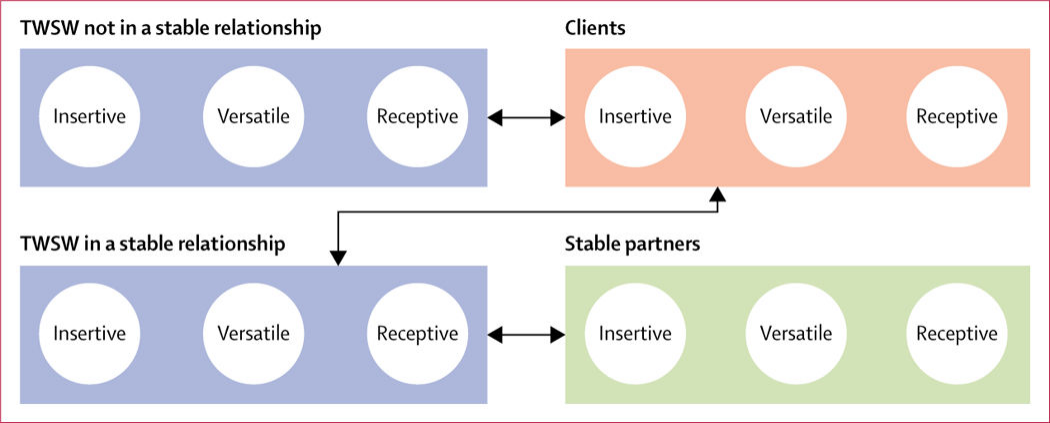
Diagram of the modelled populations and sexual mixing patterns TWSW=transgender women sex workers. Each group is disaggregated by sexual positioning in anal intercourse. Arrows represent sexual contacts between groups. Insertive=exclusively insertive. Receptive=exclusively receptive. Versatile=both insertive and receptive.

We adapted a published deterministic compartmental model to simulate HIV transmission among TWSW, their stable partners, and their clients.^9^ Briefly, the population was divided into TWSW not in a stable relationship, TWSW in a stable relationship, the stable partners of those TWSW, and the clients of all TWSW (figure 1). Stable partnerships were defined as relationships lasting on average 1 year (range 0·4–3·0). The model accounts for sexual positioning in anal intercourse (exclusively insertive, exclusively receptive, or versatile) and differences in behaviour by partner type (number of sex acts per partner and frequency of condom use). Full details on the representation of sexual behaviour, demography, and HIV transmission dynamics are provided in the appendix (pp 2–13).

### Model fitting and uncertainty

The model was fitted to time-series HIV prevalence data among transgender women in Lima using Latin hypercube sampling.^10^ The time-series prevalence data were obtained from sentinel HIV surveillance and studies carried out in Lima. Using parameters describing the natural history of HIV infection and population demographics (size and growth), we ran 100 000 parameter sets, of which 498 were within the limits for HIV prevalence. The 50 best fits according to log-likelihood were used for the analysis to give the minimum and maximum values of intervention effect for each combination of interventions. Full details are provided in the appendix. We used Matlab version R2015b for all analyses.

### Characterisation of HIV prevention interventions

To inform the implementation of interventions in the model, we designed and led group work during a national HIV prevention consultation done in November, 2014, which gathered almost 100 stakeholders representing all parties concerned (see appendix pp 21–22 for the material used in the consultation). Implementation strategies, realistic estimates of coverage, time required for scale-up, and adherence for the selected interventions were discussed, taking into consideration current national HIV care system limitations (low testing rates in particular). This input was used in conjunction with the 2017 National Quinquennial HIV Plan to design the interventions to be modelled in our scenarios.

In our basic scenario, the five interventions evaluated were (A) tailored promotion of condom use with clients, (B) tailored promotion of condom use with stable partners, (C) expansion of ART coverage to individuals with CD4 count of less than 500 cells per mm^3^, (D) expansion of ART coverage to individuals with CD4 count of at least 500 cells per mm^3^, and (E) introduction of a pre-exposure prophylaxis (PrEP) programme.

Tailored condom promotion with clients consisted of three components: (1) a condom promotion campaign, implemented yearly and developed on the basis of a market and behaviours study to tailor messages and advertising methods to TWSW; (2) condom negotiation and erotisation workshops, assumed to reach all TWSW through a so-called train the trainer method in which 10% of TWSW are trained every year and train other TWSW; and (3) a condom inundation aimed to have branded condoms and lubricant highly accessible at strategic locations. A 15% relative increase in condom use with clients after 3 years was assumed on the basis of the group work and published studies.^9,11^

Tailored condom promotion with stable partners included the same three components as above but implied separate processes and therefore spending. It was assumed to achieve a 10% relative increase in condom use after 3 years to reflect additional challenges involved in increasing condom use with stable partners compared with clients.

The first ART coverage intervention consisted of the expansion of coverage under the Peruvian guidelines effective between 2014 and 2017 (ie, to individuals with CD4 count <500 cells per mm^3^), relying on successful testing scale-up. The use of mobile outreach testing units was proposed as the most viable strategy to increase testing among TWSW because it has successfully been used in Peru.^12^ Treatment was assumed to reach a coverage of approximately 65% (vs 35% in the status quo scenario) after 5 years for the best epidemic fit with variations across fits and implemented through assuming an average testing and treatment wait of 2 years. ART effectiveness in reducing transmissibility was assumed to be 75%, which is lower than reported among heterosexual partnerships, based on lower levels of viral suppression observed among transgender women in other settings.^13^

The second ART coverage intervention consisted of expansion of coverage under the 2015 WHO guidelines (ie, irrespective of CD4 count,^14^ also known as test and offer), which were adopted in Peru in March, 2018, relying on successful testing scale-up. This was implemented as an expansion of the first ART coverage intervention, by incorporating treatment among those with a CD4 count of 500 cells per mm^3^ or more (2-year average wait for treatment) on top of the increased treatment rates implemented above, resulting in a coverage of approximately 75% and same effectiveness.

Finally, the PrEP intervention included a communication strategy, recruitment through mobile units, and clinical monitoring. It also included condom distribution, assumed to cover 50% of protected sex acts among PrEP users. A 15% coverage of the TWSW population after five years was assumed based on inputs from stakeholders. Adherence was conservatively assumed to be similar than observed in the IPrEX trial (resulting in an effectiveness of 44%) based on recent evidence from the IPrEX Ole suggesting lower adherence among transgender women.^15^

Investigation of an enhanced scenario, which included achieving the UNAIDS 90–90-90 targets^16^ among TWSW and considered improved implementation of the selected interventions, was also deemed important by stakeholders to provide concrete directions towards gold-standard HIV combination prevention. In the enhanced scenario, for condom interventions (interventions A and B), the relative increase in condom use with clients was assumed to be 20% and 15% with stable partners (versus 15% and 10% in the basic scenario, respectively) through a two-times increase (20% vs 10%) in the proportion of TWSW trained. For ART interventions (interventions C and D), to achieve the 90–90-90 UNAIDS targets, the enhanced scenario entailed (1) scale up of mobile testing units; (2) new information system to improve linkage to care post diagnosis; (3) individual linkage, retention, and adherence support programme through peer educators; (4) sensitisation of health professionals to transgender women issues; and (5) strengthening of the drug supply chain. For the PrEP intervention (intervention E), PrEP effectiveness was assumed to be 85% (*vs* 44% in the basic scenario) achieved through the implementation of an adherence support programme. A 3-year scale up (*vs* 5 year in the status quo scenario) was assumed due to higher testing rates. Both scenarios are summarised in the table.

### Costing

The costs of each intervention component were determined according to a health system perspective, using an ingredients-based approach (appendix pp 14–19). Unit costs for human resources, consumables, communication strategies, and other recurrent costs were based on government reports and peer reviewed articles or sourced directly from institutions delivering these interventions. Current building and equipment infrastructure were assumed sufficient to support the interventions considered. However, substantial improvements in the testing services (through outreach mobile units) were considered indispensable to the feasibility of the proposed interventions and, therefore, the corresponding capital and recurrent costs were included. Costs related to 2016 and were converted from Peruvian Sol to US$ based on the 2016 average currency exchange rate. Costs and benefits were discounted yearly to account for the stronger value assigned to present versus future events.

### Health outcomes and cost-effectiveness

We used disability-adjusted life-years (DALYs), composed of years of life lost and years lived with disability, to measure the negative health outcomes associated with HIV infection that could be averted through the interventions investigated; calculations of DALYs are shown in the appendix (pp 19–20).

We compared strategies to one another, ranking them from lowest cost to highest cost. Strategies that were both more costly and less effective than any other (ie, strongly dominated) were excluded. We compared each remaining strategy to the next most effective and costly by calculating the incremental cost-effectiveness ratio (ICER), corresponding to the incremental cost per additional DALY averted. Any strategy that had an ICER greater than that of a more effective strategy (ie, subject to weak or extended dominance) was excluded because a combination of other strategies would be more effective for a given cost. Finally, ICERs for the remaining strategies (ie, non-dominated) were calculated. These are plotted to form a cost-effectiveness frontier, showing which interventions provide greatest health gains for any given investment cost.

Assessing whether an ICER offered by any strategy represents value for money requires comparison to a cost-effectiveness threshold (CET). The CET represents the health effects forgone (so-called opportunity costs) associated with resources being committed to an intervention and consequentially being unavailable for other health-care priorities in Peru.^17^ Policy makers should be willing to invest limited resources in the strategy offering greatest health gains, but with the ICER remaining less than the CET.

We use CETs derived specifically for Peru by Ochalek and colleagues^18^ using statistical models to estimate the correlation between changes in health expenditure and changes in mortality and morbidity in this country. They implemented different methods and assumptions to estimate CETs, resulting in eight values. We used the lowest and highest bounds, corresponding to US$208 per DALY averted and $1300 per DALY averted, respectively. These CETs are several times lower than 1–3 times gross domestic product per capita, as previously recommended by WHO, and lower than the estimate of the Peruvian CET of $1969–7747 per DALY averted given by Woods and colleagues.^17^ We therefore adopted stringent criteria to determine cost-effectiveness.

### Role of the funding source

The funding source had no role in the study design, data collection, analysis, interpretation, or writing of the report. AB, AS-S, XS, JVG, PC, and CFC had access to the raw data. The corresponding author had full access to all the data and the final responsibility to submit for publication.

## Results

The estimated costs of the interventions are briefly described below. Individual intervention component costs are given as point estimates whereas ranges are provided for total or implementation costs reflecting uncertainty in demographic and epidemiological parameters. Costing of the condom interventions and of the ART interventions is presented once for simplicity. Full details are provided in the appendix (pp 14–20).

In the basic scenario, for condom promotion and distribution, we assumed distribution of branded condoms because stigma associated with bulk condoms was highlighted by several participants. A cost of $1·2 per condom (four times the price of bulk condoms) and a 25% loss during the distribution were assumed. The cost over 10 years of a 15% relative increase in condom use with clients was estimated at $4·0 million (range 1·8–5·4) and a 10% increase in use with stable partners at $1·0 million (0·6–1·5).

For expansion of ART coverage, the cost of antiretroviral drugs (90% generic) was estimated at $200 per person per year, whereas the cost of monitoring was estimated at $309 per person per year, giving a total of $509 per person per year, with additional costs of $124 for treatment initiation (appendix pp 15–16). The cost of pre- ART monitoring was not included. Parallel increases in diagnosis rates were assumed to be achieved through mobile units at an estimated $14·5 per HIV test (appendix pp 16–17). The cost of providing ART to TWSW with a CD4 count of less than 500 cells per mm^3^ for 10 years was estimated at $2·5 million (range 1·7–6·2) whereas ART provision independent of CD4 count was estimated at $3·0 million (2·2–8·1).

Based on the price of the branded efavirenz–emtricitabine Truvada (Gilead; Foster City, CA, USA) of $120 per person per month for individual purchase, PrEP cost was estimated at $1470 per person per year for drugs and monitoring with additional costs associated with positive diagnosis of users at recruitment, condom distribution, and a communication strategy. We also investigated a scenario using generic PrEP drugs at a much lower cost (ie, $75 per person per year) that could potentially be achieved if an agreement between the Ministry of Health and the PAHO strategic fund is signed,^19^ resulting in a total estimated cost of $105 per person per year. The cost of providing PrEP to 15% of TWSW for 10 years was estimated at $15·2 million (range 9·2–26·9) with Truvada and $1·8 million (1·1–2·8) with generic PrEP drugs.

The costs of the additional intervention components included in the enhanced scenario are provided in the appendix (pp 18–19).

**Figure 2:**
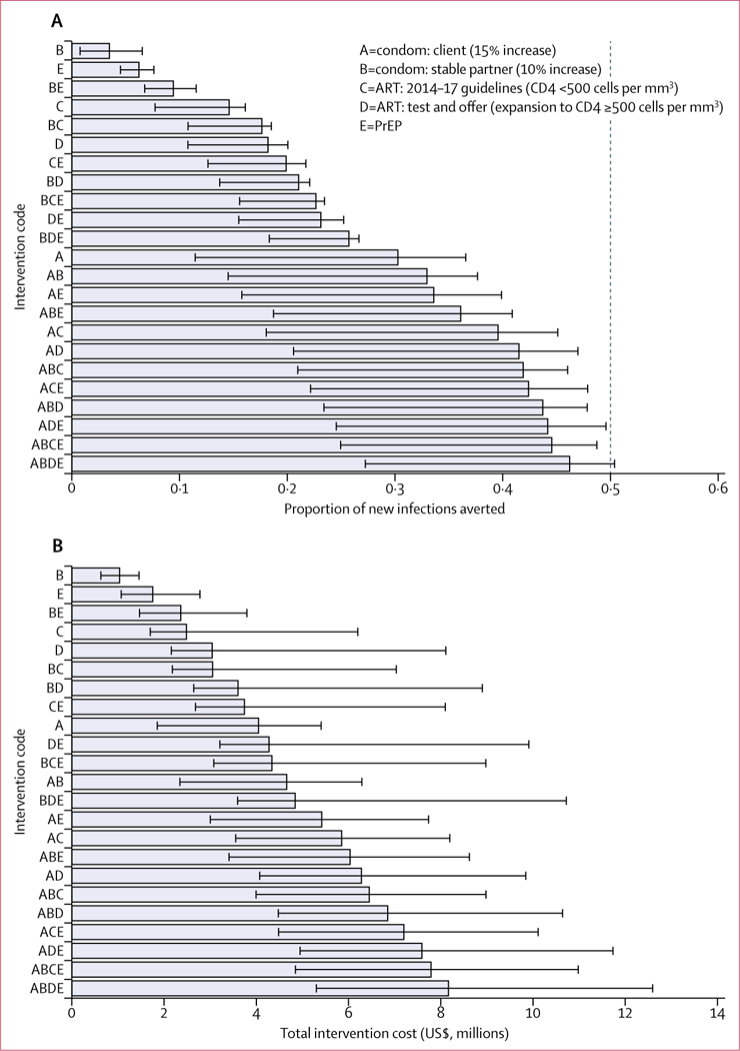
(A) Proportion of infections averted among TWSW, clients, and stable partners in Lima, Peru, with each intervention scenario and (B) total cost over 10 years for each intervention in the basic scenario The bars correspond to the best fitting simulation and the error bars correspond to the minimum and maximum estimates from the 50 best fits included in the analysis. The dashed line in panel A represents the UNGASS goal of 50% of new infections averted in the next 10 years. Intervention D includes the implementation of intervention C because it is an expansion of C. ART=antiretroviral treatment. PrEP=pre-exposure prophylaxis. TWSW=transgender women sex workers. UNGASS=UNGA Special Session on Drugs.

We estimated the impact and cost-effectiveness of all the combinations of interventions for both the basic and the enhanced scenarios and considered the effect of the different intervention combinations in terms of proportion of infections averted. In the basic scenario, between 4% (range 1–6) and 47% (27–51) of new infections occurring among TWSW and their clients and stable partners over the 10-year period could be averted through the various interventions considered (figure 2). Implementing all five interventions could potentially achieve a 50% incidence reduction, corresponding to the UNGA Special Session on Drugs (UNGASS) goal.

The total cost of implementing the full intervention package is expected to be approximately $8·2 million (range 5·3–12·6) over this period (figure 2). All interventions including PrEP under the current Truvada cost assumptions were at least twice as expensive and therefore we only present the scenario assuming generic PrEP drugs (see appendix p 23 for alternative scenario with Truvada).

**Figure 3:**
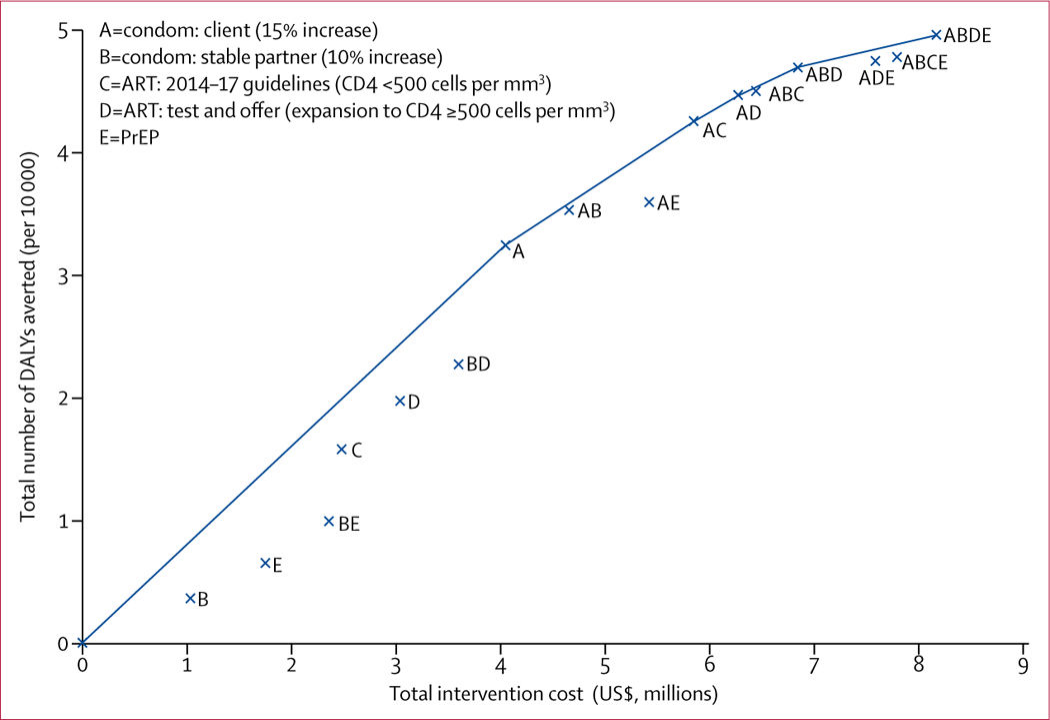
Incremental cost-effectiveness graph showing the cost and number of DALYs averted for interventions in the basic scenario The line represents the cost-effectiveness frontier and links non-dominated strategies, which provide the highest incremental health benefit for any given incremental investment cost. Weakly dominated strategies appear below the frontier. Strongly dominated strategies (ie, both more costly and less effective than any other) are not represented in the graph. Intervention D includes the implementation of intervention C because it is an expansion of C. ART=antiretroviral treatment. DALYs=disability-adjusted life-years. PrEP=pre-exposure prophylaxis.

We calculated ICERs for each of the non-dominated strategies and show the cost-effectiveness frontier (figure 3). Five strategies were non-dominated: intervention A (with an ICER of $125 per DALY averted) and intervention packages A and C ($179 per DALY averted); A and D ($202 per DALY averted); A, B, and D ($245 per DALY averted); and A, B, D, and E ($509 per DALY averted). All five of these strategies’ ICERs were under the high bound CET ($1300 per DALY averted) whereas strategies A; A and C; and A and D were under the low bound CET ($208 per DALY averted).

**Figure 4:**
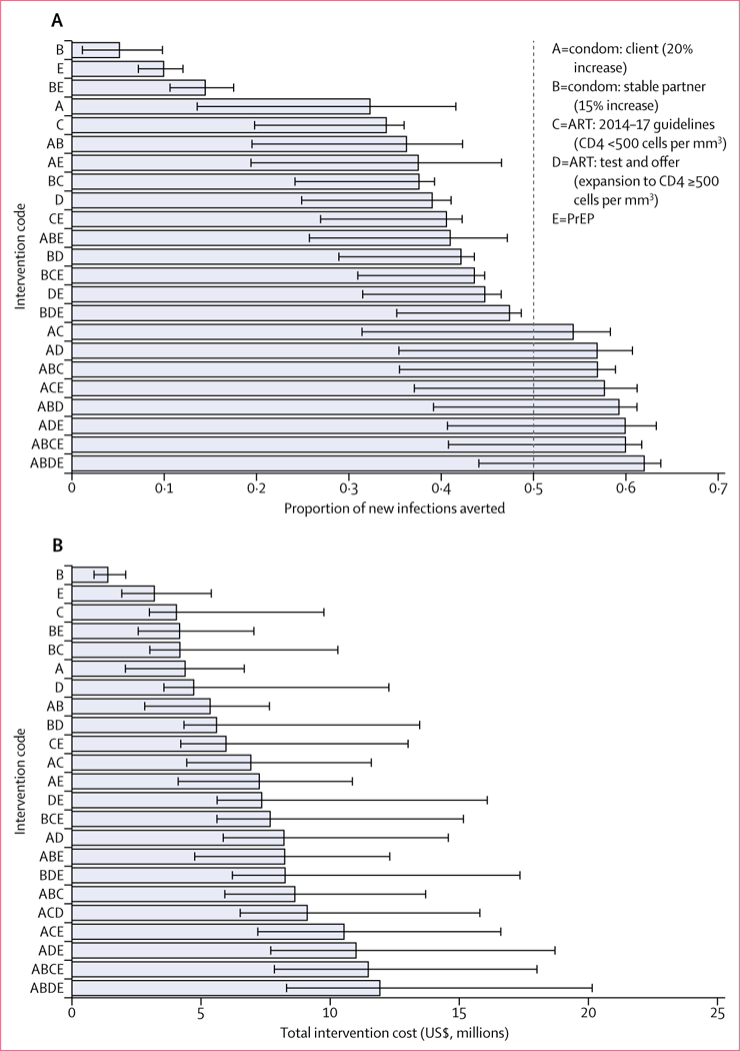
(A) Proportion of infections averted among TWSW and their clients and stable partners in Lima, Peru, and (B) total cost over 10 years for each intervention in the enhanced scenario The bars correspond to the best fitting simulation and the error bars correspond to the minimum and maximum estimates from the 50 best fits included in the analysis. The dashed line in panel A represents the UNGASS goal of 50% of new infections averted in the next 10 years. Intervention D includes the implementation of intervention C because it is an expansion of C. ART=antiretroviral treatment. PrEP=pre-exposure prophylaxis. TWSW=transgender women sex workers. UNGASS=UNGA Special Session on Drugs.

For the enhanced scenario, we considered the effect and total cost of implementing the enhanced interventions and achieving the UNAIDS 90–90-90 targets, assuming away any feasibility constraints (figure 4). Between 5% (range 1–10) and 62% (44–64) of new infections among TWSW, their clients, and their stable partners over the 10-year period could be averted under these assumptions (figure 4). The UNGASS goal of preventing 50% of new infections in the next 10 years can be achieved with a number of other combinations (intervention packages A and C; A and D; A, B, and C; A, C, and E; A, B, and D; A, D, and E; A, B, C, and E; and all five interventions; figure 4). The total cost of implementing the full intervention package under the enhanced scenario is estimated at approximately $11·9 million (range 8·3–20·1) over this period (figure 2).

**Figure 5:**
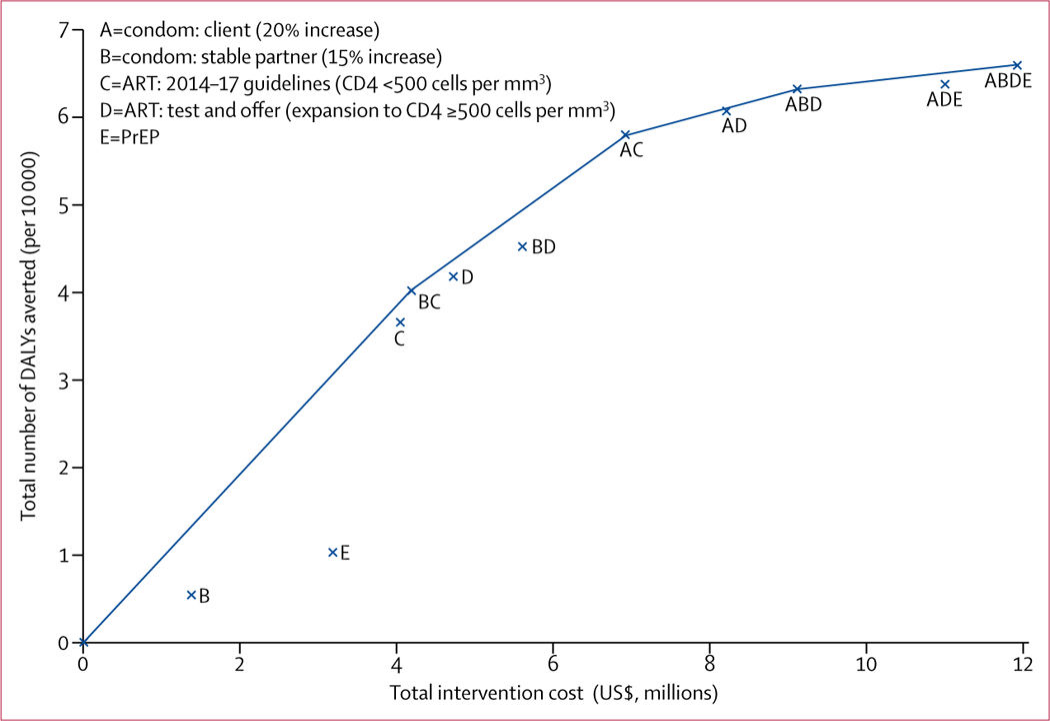
Incremental cost-effectiveness graph showing the cost and number of DALYs averted for interventions in the enhanced scenario The line represents the cost-effectiveness frontier and links non-dominated strategies, which provide the highest incremental health benefit for any given incremental investment cost. Weakly dominated strategies appear below the frontier. Strongly dominated strategies (ie, both more costly and less effective than any other) are not represented in the graph. Intervention D includes the implementation of intervention C because it is an expansion of C. ART=antiretroviral treatment. DALYs=disability-adjusted life-years. PrEP=pre-exposure prophylaxis.

Four strategies were non-dominated in the enhanced scenario (figure 5): intervention packages B and C (with an ICER of $104 per DALY averted); A and C ($155 per DALY averted); A,B, and D ($414 per DALY averted); and A, B, D, and E ($1003 per DALY averted). All of these strategies’ ICERs were below the high bound of the CET, whereas intervention package B and C and intervention package A and C were under the low bound of the CET.

## Discussion

This study provides estimates of the impact, cost, and cost-effectiveness of various HIV prevention interventions among TWSW in Lima, alone or in combination, that are considered feasible conditional on strengthening of testing services, as well as assuming enhanced HIV prevention services. Implementing test and offer, combined with tailored condom promotion with clients and stable partners and PrEP would avert close to 50% of new infections over 10 years accounting for current programme limitations. It would require an average expenditure of $816 000 per year and would be cost-effective even against a stringent CET specifically calculated for Peru ($1300 per DALY averted).

If the UNAIDS 90–90-90 targets were reached and this enhancement in HIV services also translated in more effective condom use and PrEP interventions, more than 60% of new infections among TWSW, their stable partners, and their clients would be averted. The average annual cost would be approximately $1·2 million and this scenario would also be cost-effective. Implementation of this strategy would represent approximately 2% of the total national annual spending on HIV/AIDS for Peru (which was $60 million in 2014) and 17% of the spending allocated to key populations (which was $7 million in 2012, although this figure does not include treatment).^20^ It also represents about 10% of the Global Funds’ current annual contribution in Peru ($12 million in 2018), which is almost exclusively spent on prevention among key populations. The decision of which of these combinations is likely to represent best use of resources should consider appropriateness and feasibility and not solely be based on the ICER, because ICERs carry uncertainty and are very similar for some combinations. However, our results indicate that the expenditure required from the health authorities in Peru to implement the full intervention package with enhanced HIV services is justified given the health gains.

Increasing condom use remains challenging. The design of the condom intervention in our study addresses the issue of condom fatigue through the inclusion of intensive, innovative—and costly—condom interventions. Workshops that assist TWSW in negotiating condom use during commercial sex have been shown to effectively increase use to higher levels than assumed here.9 However, the model assumes the increase would be sustained over 10 years through periodic intervention implementation, which needs to be shown. Additionally, the model does not capture the fact that some TWSW might be unable to increase their condom use, which would result in pockets of higher transmission possibly affecting overall intervention impact.

Increases in TWSW condom use with stable partners had a much lower impact in both the basic and enhanced scenarios. This is due to the low proportion of TWSW in a stable partnership and to the lower increase in condom use assumed. Increasing condom use with stable partners has proven challenging among female sex workers^21,22^ and qualitative work indicates this also to be true among TWSW in Lima. No data are available on stable partners of TWSW in Lima. In other settings, stable partners of TWSW have been shown to have multiple partners of either sex and high use of alcohol and drugs, suggesting they have a key role in transmission within the MSM and transgender women population and beyond.^23,24^ Promoting condom use and alternative strategies such as PrEP with these partners should therefore remain a priority.

Treatment according to the 2014–17 Peruvian guidelines and the test-and-offer strategy were equivalent in terms of cost-effectiveness in both scenarios. Indeed, our study contributed evidence supporting the change in treatment guidelines in Peru effective March, 2018, recommending ART independent of CD4 count. However, implementation of these guidelines relies on high increases in testing rates. Reduced investment in sexual health clinics has led to the services provided being unsuitable for many transgender women due to inflexible opening hours, long waiting times, untrained staff, and a lack of outreach services. Additionally, a disjointed HIV care system hampers the linkage of newly diagnosed patients to care and impedes the systematic monitoring of the care continuum.^5^ We incorporated and costed intervention components that address these issues.

Low treatment adherence has been documented among transgender women in other settings including San Francisco,^13^ where the quality of HIV care is high. It is associated with stigma and discrimination in health-care services, fears of the treatment interfering with hormonal therapies, unstable living conditions exacerbated by the use of alcohol and drugs, and psychological issues.^9,13^ In Peru, this is aggravated by ART shortages. In our enhanced scenario, we included and costed complementary intervention components to reach the 90–90-90 UNAIDS targets. Ideally, HIV care should be integrated with feminisation and psychological treatments, which we did not incorporate in this study but has recently been explored in Peru.^25^

Inclusion of PrEP in the intervention package adds to impact but requires generic drug prices to be cost-effective. Generic PrEP (ie, Mylena) is now available through some providers in Peru and through a PrEP demonstration project (ImPrEP) ongoing in six cities among MSM and among transgender women. It could be available at large scale through the Ministry of Health conditional on an agreement with the PAHO Strategic Fund. We used conservative estimates for PrEP effectiveness as compared with those observed in the PROUD^26^ and Ipergay^27^ studies due to the lower adherence observed among transgender women in the PrEP Ole study,^15,28^ suggesting this population requires additional and better tailored adherence support. We included this in our enhanced scenario; however, it is likely that in real-world conditions, users will self-select and adherence will be higher. Our study is in line with other modelling studies that have found PrEP to be cost-effective among transgender women.^29,30^

The use of a mixed-methods study to inform intervention design was an innovative and productive endeavour in the context of HIV prevention policy among TWSW in Peru and contributed to the development of the first plan for comprehensive HIV prevention and care for transgender women in Peru.^31^ We believe this approach should be encouraged for the planning of effective intervention programmes more widely. However, it comes with challenges and limitations. First, we rely on the model’s ability to accurately reproduce HIV epidemic dynamics among TWSW and their partners. We have endeavoured to represent main drivers of risk among this population, including different types of partners and key sexual behaviors and have accounted for the underlying uncertainty in parameter values by presenting results for a range of epidemic trajectories that fit the available data. We note that we only considered sexual transmission; although injection drug use is rare in Lima, transgender women have reported injecting in the context of body modification procedures (eg, collagen or silicone injections into the glutes, hips, breasts, forehead, and chin).^32^ If syringe sharing occured in this context it could potentially translate in an overestimation of the interventions’ impact. Second, integrating information from stakeholder analyses to the model requires dealing with subjectivity and differences in opinions. To address this when determining parameter ranges for the interventions, we did group work and discussed outcomes among a larger group to contrast and validate results. To respond to different demands to inform policy planning (ie, acknowledge current system limitations and set aspirational goals), we implemented scenario analyses. Third, the design—and therefore the costing—of the interventions is subject to discussion and uncertainties. However, we based it on local studies, including our health system capacity assessment.

Our study has important strengths. The modelling analysis was integrated from the conception of the mixed-methods study, ensuring specific questions to inform the model were clearly formulated and included in the design of each component. It was based on a dialogue with stakeholders and is therefore responsive to their concerns and needs, increasing its relevance for policy planning. Indeed, it could be used beyond the HIV programme-planning phase, to monitor and evaluate the implementation of HIV interventions among TWSW. In turn, this would allow validating assumptions regarding their effectiveness under specific implementation conditions. Involving stakeholders in the application of mathematical modelling studies is feasible and contributes to the development of more relevant cost-effectiveness analyses to support programmatic decision-making.

## Supplementary Material

1

## Figures and Tables

**Table :** Code and definition of interventions implemented in the model and corresponding magnitude of change, effectiveness, coverage, and time to scale-up, for the basic and enhanced scenarios

	Magnitude	Effectiveness	Coverage	Time to scale-up
**Higher condom use with clients**				
Basic	15% relative increase	70%	100%	3 years
Enhanced	20% relative increase	70%	100%	3 years
**Higher condom use with stable partners**				
Basic	10% relative increase	70%	100%	3 years
Enhanced	15% relative increase	70%	100%	3 years
**ART 2014–17 guidelines**				
Basic	CD4 count <500 cells per mm^3^	75%	65%	5 years
Enhanced	CD4 count <500 cells per mm^3^	90%	90%	5 years
**Test and offer**				
Basic	CD4 count ≥500 cells per mm^3^	75%	75%	5 years
Enhanced	CD4 count ≥500 cells per mm^3^	90%	90%	5 years
**PrEP**				
Basic	Suboptimal adherence	44%	15%	5 years
Enhanced	Good adherence	85%	15%	3 years

ART=antiretroviral treatment. PrEP=pre-exposure prophylaxis.

## Research in context

### Evidence before this study

Transgender women have a consistently high risk of HIV in a range of settings, with a pooled HIV prevalence estimated at 19·1% (95% CI 17·4–20·7), corresponding to a nearly 50-times greater odds of infection relative to the general population. Designing effective HIV prevention programmes tailored to this population is therefore a priority. We reviewed the literature to identify mathematical modelling studies investigating HIV prevention among transgender women by searching for the following terms in PubMed on May 24, 2018, with no date or language restrictions: (“HIV”) AND (“prevention”) AND (“transgender” OR “transwomen”) AND (“model*”). Three mathematical modelling studies were identified. One study investigated the cost-effectiveness of pre-exposure prophylaxis (PrEP) among men who have sex with men (MSM) and transgender women in Lima, Peru, following the IPrEX trial and found that PrEP would be a cost-effective intervention if prioritised to transgender women at higher risk for HIV using both the less than 1 GDP per capita criterion and the more stringent World Bank criterion. Another study addressing this same question among transgender women in Brazil using a non-dynamic mathematical model found that PrEP would decrease lifetime HIV infection risk among high-risk MSM and transgender women from 50·5% to 40·1% and increase per-person life expectancy from 36·8 years to 41·0 years and would be highly cost-effective using the less than 1 gross domestic product per capita criterion. One study considered different combinations of interventions among transgender women sex workers (TWSW) in Lima, Peru, and San Francisco, CA, USA, and found that achieving a 50% reduction in incidence over 10 years was possible in both settings, but required different combinations of interventions as underlying behaviours and HIV service coverage among transgender women differ substantially. This study did not investigate cost-effectiveness.

### Added value of this study

Our study investigated impact and cost-effectiveness of different combinations of interventions for TWSW in Lima, Peru. It was embedded in a broader mixed-methods study aiming to develop a reproducible process to design tailored HIV prevention programmes among MSM and transgender women, using Peru as a test case. The need to specifically model transgender women and to consider their high exposure to sex work emerged from the stakeholder analysis. We found that a combination of interventions increasing condom use with clients by a relative 15% and with stable partners by 10% as well as 15% PrEP coverage among transgender women and scale-up of antiretroviral treatment (ART) to reach approximately 75% of HIV-positive transgender women could prevent nearly 50% of new infections among this population over a 10-year period and would be cost-effective according to stringent criteria specific to Peru. The study also indicated that further investment to reach the UNAIDS 90–90-90 targets and enhance condom use and PrEP interventions would surpass the 50% incidence reduction goal and remain cost-effective.

### Implications of all available evidence

Mathematical modelling has shown that investing in HIV prevention (including ART as prevention) among transgender women is cost-effective. Challenges in condom use, HIV testing access and uptake, and ART and PrEP linkage and adherence among this population need to be considered at the design stage of HIV prevention programmes and included in cost-effectiveness analyses to provide realistic cost estimates. This study is relevant to other settings in Latin America and more broadly, because transgender women around the world face similar vulnerabilities that increase their risk of HIV; this risk should be addressed through HIV combination prevention within an integrated health model.
