# Supplementary material for: The impact and cost-effectiveness of combined HIV prevention scenarios among transgender women sex-workers in Lima, Peru: a mathematical modelling study
Source: Lancet Public Health. Author manuscript; Available in PMC 2019 Apr 3. (PMC6447307; doi:10.1016/S2468-2667(18)30236-6)
Supplement: 1 [file NIHMS1523388-supplement-1.pdf]

THE LANCET
Public Health

Supplementary appendix

This appendix formed part of the original submission and has been peer reviewed. We post it as supplied by the authors.

Supplement to: Bórquez A, Guanira JV, Revill P, et al. The impact and cost-effectiveness of combined HIV prevention scenarios among transgender women sex-workers in Lima, Peru: a mathematical modelling study. *Lancet Public Health* 2019; published online Jan 22. http://dx.doi.org/10.1016/S2468-2667(18)30236-6.

Technical Appendix

THE IMPACT AND COST-EFFECTIVENESS OF COMBINED HIV PREVENTION SCENARIOS AMONG TRANSGENDER WOMEN SEX-WORKERS IN LIMA, PERU: A MATHEMATICAL MODELLING STUDY

Bórquez Annick,[1,2] PhD; Guanira Juan Vicente,[3] MPH; Revill Paul[4]; PhD, Caballero Patricia,[5-6] PhD; Silva-Santisteban Alfonso,[3] MD; Kelly Sherrie,[7] PhD; Salazar Ximena,[3] PhD; Bracamonte Patricia,[8] MPH; Minaya Percy,[6] MD; Professor Hallett Timothy B.,[1] PhD; Professor Cáceres Carlos F.[3], PhD.

1. Imperial College London, UK; 2. University of California San Diego, US; 3. Universidad Cayetano Heredia, Peru; 4. University of York, UK; 5. Instituto Nacional de Salud, Peru; 6. Ministry of Health, Peru; 7. Burnet Institute, Australia; 8. UNAIDS, Peru.

Table of contents

1. Model 2
Sexual behaviour 2
Demography 2
HIV natural history and treatment 3
Equations 3
Partnership formation and dissolution among TW-SW 5
Force of infection 6
Mixing matrix 7
2. Model fitting: parameter values and sources 10
3. Costing 14
PrEP intervention 14
HIV treatment interventions 15
Screening with mobile units 16
Condom promotion 18
4. Calculation of DALYs averted per HIV infection averted 19
5. Cost effectiveness 21
6. Stakeholder analysis and health system capacity assessment 21
Stakeholder analysis 21
Health system capacity assessment 22
7. Cost and incremental cost-effectiveness of interventions when assuming the branded (Truvada®) efavirenz/emtricitabine of USD 120/person/month 23

1. Model

Sexual behaviour

Using a published deterministic model we simulated HIV transmission among TW-SW, their clients and stable partners.[1] The population was divided into TW-SW in a stable relationship, other TW-SW, clients, and stable partners of TW-SW. Stable partnerships were defined as relationships lasting 1 year on average to account for short term partnerships of a few months as well as long term partnerships. Only qualitative data was available to inform this parameter so it was given broad variability in the uncertainty analysis. The model accounts for sexual positioning (exclusively insertive, exclusively receptive or versatile) and differences in behaviours by partner type (number of sex acts/partner, condom use levels and sexual positioning) among TW-SW.

Condom use was assumed to increase linearly after 1985 for a period of 10 years and to stay stable thereafter. It was assumed to reach 75% with clients and under 50% with stable partners based on condom use reports. Its efficacy was estimated at 70% based on the most recent systematic review among MSM in the US.[2]

Demography

The size of the TW population in Lima is estimated at 20,000, and about 65% of these report being sex workers (13,000).[3] According to the most recent study available, 25% reported being in a stable relationship[3] translating in a population of partners of about 3,250 men. Based on their reported numbers of clients per week as well as on the estimated population of MSM in Lima we estimated the client population to be of about 114,000. Population growth was assumed to be constant at 2% and the distribution of the population by group was assumed to remain constant through time. Life expectancy among TW-SW was estimated at 60 years versus 70 years among stable partners and clients based on very low estimates of life expectancy among TW in Latin America and higher HIV prevalence among MSM respectively.

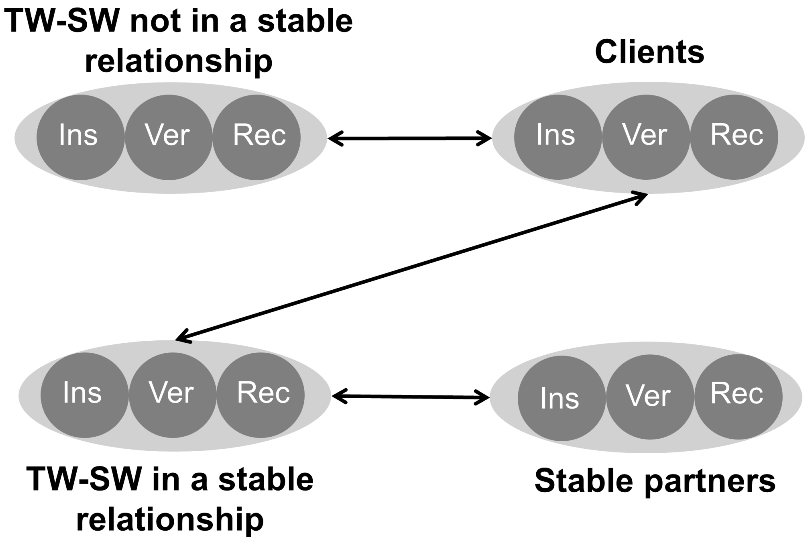

Figure 1. Model diagram showing the sexual contacts between the populations included

HIV natural history and treatment

HIV progression and treatment was represented by 11 stages (figure 2), including acute infection of short duration and high infectivity, early latent infection with CD4>500 cells/mm^3, medium latent infection with 500-350 CD4 cells/mm^3, late latent infection with 350-200 CD4 cells/mm^3, with lower infectivity and a total duration of approximately 8 years; Pre-AIDS for <200 CD4 cells/mm^3 of higher infectivity and duration of approximately 1 year and AIDS with no infectivity due to illness.[4,5]
ART treatment started in 2002 and increased linearly to reach a coverage of 80% of HIV positive individuals with <200 CD4 cells/mm^3 in 2007.[6] Treatment to HIV positive individuals with: 350-200 CD4 cells/mm^3 was implemented in the model in 2012 to reflect the change in national guidelines. The rate of treatment was manually fit to the estimated coverage in 2010. Based on estimates from the Peruvian National Institute of Health, the proportion of diagnosed individuals linked to ART care was 95% if diagnosed with CD4 count below 200 cells/mm^3 and 90% if diagnosed with a CD4 count between 200 and 350 cells/mm^3. The yearly dropout rate was 0.005/year and 0.01/year for those with a <200 CD4 cells/mm^3 and between 200 and 350 cells/mm^3 respectively. As observed elsewhere, the data suggested that lower CD4 counts were associated with higher linkage to care and lower dropout rates.
The Peruvian treatment guidelines changed in December 2015 to include patients with a CD4 count <500 cells/mm^3 and our study also considered treatment for those with CD4 counts >500 cells/mm^3, reflecting the 2015 WHO treatment guidelines (adopted in Peru in March 2018). For those entering ART at CD4 counts between 350 and 500 cells/mm^3 and >500 cells/mm^3, linkage to care was assumed at 80% of those diagnosed and the dropout rate at 0.015/year. The rate of treatment was set at 0.5, corresponding to testing (and receiving treatment if infected) every two years on average, and achieved over 5 years, as this was considered a feasible goal. In the enhanced scenario this rate was increased to 0.75, corresponding to testing every 1.3 years on average. The ART interventions also assumed a treatment rate of 0.5 (and 0.75 for the enhanced scenario) among those with CD4 counts between 200-350 cells/ mm^3 for consistency.

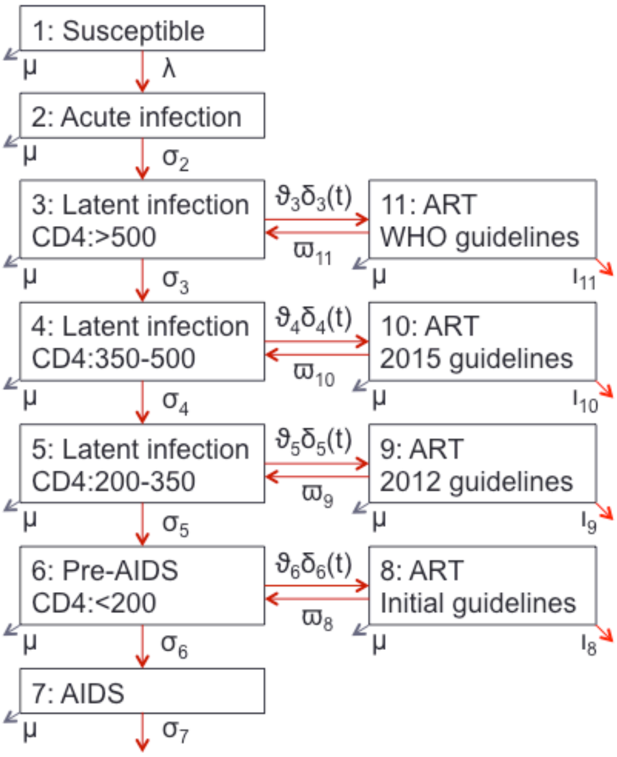

Figure 2. Representation of HIV natural history and treatment in the model

Equations

We modified and updated a published model to implement this study. The model is defined by ordinary differential equations to simulate how HIV spreads over time. These are shown below. The state variables are given by $X_k^s(t)$. t is the time elapsed in the simulation; s is the infection-status (1= susceptible; 2= acute infection; 3= >500 CD4; 4=350-500 CD4; 5=200-350 CD4; 6= <200 CD4; 7= AIDS; 8=ART start<200 CD4; 9=ART start 200-350 CD4; 10=ART start 350-500 CD4; 11=ART start >500 CD4), k is 'sexual behaviour' (1= Insertive TW-SW; 2=Versatile TW-SW; 3=Receptive TW-SW; 4=Insertive TW-SW-SP; 5=versatile TW-SW-SP; 6=Receptive TW-SW-SP; 7=Insertive Clients; 8=Versatile

Clients; 9= Receptive clients; 10=Insertive Stable Partners; 11=Versatile Stable Partners; 12=Receptive Stable Partners. The distribution by sexual behavior is determined by parameter κ_k.

Susceptible individuals X_k^1 get infected at a rate λ_k, namely the force of infection, which is dependent on the sexual behaviour (k) (eq. 3). Acutely infected individuals progress through the infection stages characterized by decreasing CD4 counts at rates σ_2 to σ_5 as shown in equations 5 to 8, until they progress to AIDS at a rate σ_6 and death at a rate σ_7 as shown in equations 8 and 10. HIV infected individuals receive ART at different CD4 count stages depending on treatment guidelines and coverage at time t. Before 2012, only those with a CD4 count<200 cells/mm3 received ART (eq 11). Between 2012-2015 those with a CD4 count<350 cells/mm3 were eligible for ART (eq. 12). In December 2015, those with a CD4 count<500 became eligible for ART (eq. 13), and treatment independent of CD4 count (i.e. CD4 count>500) as recommended by the new WHO guidelines is also considered (eq. 14). Rates of progression to treatment from the different CD4 count stages were defined as $\delta_{s,t,k}$ where s corresponds to the infection stage, t to time and k to sexual behaviour. To account for the loss to follow up between diagnosis and treatment initiation, the rate of progression was multiplied by ϑ_s corresponding to the proportion successfully linked to treatment. Treatment drop out was implemented by a progression back at a rate ϖ_s into the CD4 stage in which ART was initiated. An additional mortality rate on ART, ι_s, corresponding to mortality due to associated causes, varies according to CD4 count at the time of ART initiation. TW-SW progress in between the TW-SW (single) and the TW-SW-SP (in a stable partnership) compartments according to M_k to represent partnership formation and dissolution, this is explained in detail in the next section.

$$G_k = (\mu + \alpha(t)) \sum_s \left(X_k^s\right) \qquad (1)$$

G corresponds to the growth of the population and is modelled through replacing all background deaths, with μ being the natural death rate, and adding a growth rate α. All individuals enter the population as susceptible to infection.

$$T_k = \tau_k \sum_s X_k^s \qquad (2)$$

T corresponds to those exiting the population through a turnover rate τ and that need to be replaced to obtain a constant population distribution. The turnover rate is independent from the infection stage but specific to each sexual behaviour group k.

$$A_k = \sigma_7 \cdot X_k^7 + \iota_8 X_k^8 + \iota_9 X_k^9 + \iota_{10} X_k^{10} + \iota_{11} X_k^{11} \qquad (3)$$

A corresponds to the HIV/AIDS and ART associated deaths that need to be replaced to obtain the desired population growth. σ_7 is the progression rate from AIDS to death and ι_8 to ι_{11} are the death rates from HIV associated causes among those on ART based on the CD4 count at treatment initiation

All background deaths and exits associated to natural population turnover and HIV/AIDS in each sexual behaviour group are replaced in the susceptible stage of the corresponding group so that the population distribution by sexual behaviour group is maintained constant through time as shown in equation 4 below.

$$\frac{dX_k^1(t)}{dt} = \left\{G_k + \mathrm{T}_k + A_k\right\} - \lambda_k X_k^1 - (\mu + \tau_k)X_k^1 \ + \mathrm{M_k} \qquad (4)$$

$$\frac{dX_k^2(t)}{dt} = \lambda_k X_k^1 - \left(\sigma_2 + \mu + \tau_k\right)X_k^2 \ + \mathrm{M_k} \qquad (5)$$

$$\frac{dX_k^3(t)}{dt} = \sigma_2 X_k^2 - \left(\sigma_3 + \mu + \tau_k\right)X_k^3 - \vartheta_3\delta_{3,k}\left(t\right)X_k^3 + \varpi_{11}X_k^{11} + \mathrm{M_k} \qquad (6)$$

$$\frac{dX_k^4(t)}{dt} = \sigma_3 X_k^3 - \left(\sigma_4 + \mu + \tau_k\right)X_k^4 - \vartheta_4\delta_{4,k}\left(t\right)X_k^4 + \varpi_{10}X_k^{10} + \mathrm{M_k} \qquad (7)$$

$$\frac{dX_k^5(t)}{dt} = \sigma_4 X_k^4 - \left(\sigma_5 + \mu + \tau_k\right)X_k^5 - \vartheta_5\delta_{5,k}\left(t\right)X_k^4 + \varpi_9 X_k^9 + \mathrm{M_k} \qquad (8)$$

$$\frac{dX_k^6(t)}{dt} = \sigma_5 X_k^5 - \left(\sigma_6 + \mu + \tau_k\right)X_k^6 - \vartheta_6\delta_{6,k}\left(t\right)X_k^6 + \varpi_8 X_k^8 + \mathrm{M_k} \qquad (9)$$

$$\frac{dX_k^7(t)}{dt} = \sigma_6 X_k^6 - \left(\sigma_7 + \mu + \tau_k\right)X_k^7 + \mathrm{M_k} \qquad (10)$$

$$\frac{dX_k^8(t)}{dt} = \vartheta_6\delta_{6,k}\left(t\right)X_k^6 - \varpi_8 X_k^8 - \left(\iota_8 + \mu + \tau_k\right)X_k^8 + \mathrm{M_k} \qquad (11)$$

$$\frac{dX_k^9(t)}{dt} = \vartheta_5\delta_{5,k}\left(t\right)X_k^5 - \varpi_9 X_k^9 - \left(\iota_9 + \mu + \tau_k\right)X_k^9 + \mathrm{M_k} \qquad (12)$$

$$\frac{dX_k^{10}(t)}{dt} = \vartheta_4\delta_{4,k}\left(t\right)X_k^4 - \varpi_{10}X_k^{10} - \left(\iota_{10} + \mu + \tau_k\right)X_k^{10} + \mathrm{M_k} \qquad (13)$$

$$\frac{dX_k^{11}(t)}{dt} = \vartheta_3\delta_{3,k}\left(t\right)X_k^3 - \varpi_{11}X_k^{11} - \left(\iota_{11} + \mu + \tau_k\right)X_k^{11} + \mathrm{M_k} \qquad (14)$$

Partnership formation and dissolution among TW-SW

In order to reproduce partnership formation and dissolution, TW-SW-SP leave the compartment at a rate ε corresponding to 1 over the average duration of a partnership υ. In order to keep the proportion in each group constant through time, partnership formation among TW-SW is a function of the partnership dissolution rate and the relative proportion in each group (TW-SW-SP/TW-SW), designed by o. Insertive TW-SW-SP (k=4) move to the insertive TW-SW (k=1) compartment and so forth as shown in equation 28 and the rates of partnership formation and dissolution are assumed to be the same independently of sexual role or infection stage.

Rate of partnership dissolution among TW-SW-SP:
This rate is equal to zero for all groups except TW-SW-SP (k=4 to 6) as shown in equations 15 to 17.

$$\varepsilon_{1..3} = 0 \qquad (15)$$

$$\varepsilon_{4..6} = 1/\upsilon \qquad (16)$$

$$\varepsilon_{7..12} = 0 \qquad (17)$$

Rate of partnership formation among TW-SW:
This rate is equal to zero for all group except TW-SW (single) (k=1 to 3). It is equal to the rate of partnership dissolution in the corresponding sexual role group among TW-SW-SP ε_k (k=1 move to k=4, k=2 move to k=5 and k=3 move to k=6) and adjusted proportionally to the size of the two groups according to κ_k as shown in equation 18 to 21.

$$o_1 = \varepsilon_4 \cdot \frac{\kappa_4}{\kappa_1} \qquad (18)$$

$$o_2 = \varepsilon_5 \cdot \frac{\kappa_5}{\kappa_2} \qquad (19)$$

$$o_3 = \varepsilon_6 \cdot \frac{\kappa_6}{\kappa_3} \qquad (20)$$

$$o_{4..12} = 0 \qquad (21)$$

Model implementation:
At each time step TW-SW-SP exit their group at a rate ε_k and TW-SW exit their group at a rate o_k as shown above. To have them entering the appropriate group, parameters $o1_k$ to $o3_k$ and $\varepsilon1_k$ to $\varepsilon3_k$ are included in the equation. These are equal to the exit rate of the origin group when k corresponds to the destination group and equal to zero otherwise. For instance $o1_4$ is equal to o_1, which allows individuals coming from group 1 to enter into group 4. The term M_k described in equation 28 is present in all ODEs and contains all exit and entry terms described above, which are turned on and off depending on the value of k.

$$o1_4 = o_1 \qquad (22)$$

$$o2_5 = o_2 \qquad (23)$$

$$o3_6 = o_3 \qquad (24)$$

$$\varepsilon1_1 = \varepsilon_4 \qquad (25)$$

$$\varepsilon2_2 = \varepsilon_5 \qquad (26)$$

$$\varepsilon3_3 = \varepsilon_6 \qquad (27)$$

$$M_k = -\varepsilon_k X_k + \varepsilon1_k X_4 + \varepsilon2_k X_5 + \varepsilon3_k X_6 - o_k X_k + o1_k X_1 + o2_k X_2 + o3_k X_3 \qquad (28)$$

The initial conditions of the system are:

$$X_k^1(0) = (1-\Omega)\kappa_k N_0 \qquad (29)$$

$$X_k^3(0) = \Omega \kappa_k N_0 \qquad (30)$$

N_0 is the size of the total population at the start of the simulation, κ_k determined the distribution of the population by sexual behavior and Ω is the HIV prevalence at the start of the simulation in all groups. Individuals are assumed to be either susceptible or in the early phase of infection at start. All other stages of infection are set at zero.

Force of infection

The force of infection determines the rate of progression from susceptible to infected. The force of infection through sexual contact depends on: the number of insertive and receptive partnerships C^i and C^r, respectively, the pattern of sexual partnership formation with respect to sexual behaviour (i.e. the proportion of partnerships formed with each of the sexual behaviour groups for insertive and receptive partnerships ($\rho^i_{k,k'}$ and $\rho^r_{k,k'}$ respectively), the number of sex acts occurring within that partnership ($\omega_{k,k'}$), the infection-status and stage of infection of the partner, the fraction of sex acts in which a condom is used ($\phi_{k,k'}$) and the efficacy of condoms in reducing the risk of HIV transmission (ψ). Condom use increases linearly for a period starting shortly after the first AIDS case was diagnosed to reproduce the increase in condom use observed among the MSM/Trans population as a result of awareness of the disease and of its transmission through sex.[33] β^i_s and β^r_s are the probabilities of HIV transmission per sex act for each stage of infection for insertive and receptive anal sex respectively. For individuals using PrEP, it will also depend on their adherence to the PrEP regimen, which determines the proportion of sex acts protected by PrEP (η_a) and on the efficacy of PrEP (θ).
As the probability of transmission varies by stage of infection, the prevalence of HIV among each partner of sexual behaviour group k' defined as $H^s_{k'}$ is calculated for each infectious stage s as shown in equation 31 and multiplied by

the corresponding risk of acquiring infection defined as R_s^i and R_s^r, when taking the insertive and the receptive role respectively. As shown in equations 32 and 33, this is equal to 1 minus the probability of not getting infected in $\omega_{k,k'}$ sex acts of which $\varphi_{k,k'}$ are protected by using a condom and therefore have a lower transmission probability, determined by ψ. The rate of sex acts in a partnership is different for commercial and stable partnerships. Likewise, the frequency of condom use is different between commercial and stable partnerships.

$$H_{k'}^s = \frac{X_{k'}^s}{\sum_s X_{k'}^s} \qquad (31)$$

$$R_s^i = \left(1 - \left(\left(1-\beta_s^i\right)^{\omega_{k,k'}\left(1-\varphi_{k,k'}\right)}\left(1-\beta_s^i\psi\right)^{\omega_{k,k'}\varphi_{k,k'}}\right)\right) \qquad (32)$$

$$R_s^r = \left(1 - \left(\left(1-\beta_s^r\right)^{\omega_{k,k'}\left(1-\varphi_{k,k'}\right)}\left(1-\beta_s^r\psi\right)^{\omega_{k,k'}\varphi_{k,k'}}\right)\right) \qquad (33)$$

The force of infection is given in equations 34.

$$\lambda_k = \sum_{k'} C_k^i \rho_{k,k'}^i \left(\sum_{s=2}^{8} H_{k'}^s R_s^i\right) + \sum_{k'} C_k^r \rho_{k,k'}^r \left(\sum_{s=2}^{8} H_{k'}^s R_s^r\right) \qquad (34)$$

Mixing matrix

The mixing matrix designs the proportion of partnerships that are formed with individuals from each of the groups. Exclusively insertive individuals cannot have sex with other exclusively insertive individuals and the same applies to receptive individuals. All TW-SW are assumed to have the same number of clients independently of whether they have a stable partner or not and independently of their sexual role. Likewise, all clients have the same number of TW-SW partners irrespective of their sexual role.

The distribution of sexual roles among the clients mirrors that of TW-SW (ie. if 80% of TW-SW are receptive then 80% of clients are insertive). This is a simplified way of having TW-SW's sexual role dependent on demand. The number of partners among clients is calculated so that the total number of partners among TW-SW and clients is the same. TW-SW are assumed to have one stable partner and the latter are assumed to have casual partners in addition to this primary relationship. TW-SW also report having casual partners aside from their primary relationship but, for simplicity, these are assimilated to the client group based on higher reported levels of condom use with these partners, as mentioned previously. Rather than explicitly modeling casual partners of stable partners of TW-SW for which there is little information available, these contacts are represented by multiplying the force of infection among stable partners by a constant that is allowed to vary in the fitting process. This increases prevalence among stable partners following their baseline epidemic trend and therefore reproduces the increased risk of exposure among TW-SW who have a stable partner. To represent the fact that TW-SW report more receptive sex with stable partners, TW-SW-SP who are versatile are assumed to always be receptive with their stable partner.

Mixing insertive TW-SW

$$\rho_{1,8} = \frac{\kappa_8 \cdot 0.5}{\kappa_8 \cdot 0.5 + \kappa_9} \qquad (35)$$

$$\rho_{1,9} = \frac{\kappa_9}{\kappa_8 \cdot 0.5 + \kappa_9} \qquad (36)$$

Mixing versatile TW-SW

$$\rho_{2,7} = \frac{\left(1 - \rho_{3,7}\right) \cdot \kappa_7}{\sum_{k=7}^{9} \kappa_k} \cdot \frac{\sum_{k=1}^{3} \kappa_k}{\kappa_2} \qquad (37)$$

$$\rho_{2,8} = 1 - \left(\rho_{2,7} + \rho_{2,9}\right) \qquad (38)$$

$$\rho_{2,9} = \frac{\left(1 - \rho_{3,9}\right) \cdot \kappa_9}{\sum_{k=7}^{9} \kappa_k} \cdot \frac{\sum_{k=1}^{3} \kappa_k}{\kappa_2} \qquad (39)$$

Mixing receptive TW-SW

$$\rho_{3,7} = \frac{\kappa_7}{\kappa_8 \cdot 0.5 + \kappa_7} \qquad (40)$$

$$\rho_{3,8} = \frac{\kappa_8 \cdot 0.5}{\kappa_8 \cdot 0.5 + \kappa_7} \qquad (41)$$

Mixing insertive TW-SW-SP

$$\rho_{4,8} = \rho_{1,8} - \frac{\rho_{1,8}}{C_4} \qquad (42)$$

$$\rho_{4,9} = \rho_{1,9} - \frac{\rho_{1,9}}{C_4} \qquad (43)$$

$$\rho_{4,12} = \frac{1}{C_4} \qquad (44)$$

Mixing versatile TW-SW-SP

$$\rho_{5,7} = \rho_{2,7} - \frac{\rho_{2,7}}{C_5} \qquad (45)$$

$$\rho_{5,8} = \rho_{2,8} - \frac{\rho_{2,8}}{C_5} \qquad (46)$$

$$\rho_{5,9} = \rho_{2,9} - \frac{\rho_{2,9}}{C_5} \qquad (47)$$

$$\rho_{5,11} = \frac{1}{C_5} \qquad (48)$$

Mixing receptive TW-SW-SP

$$\rho_{6,7} = \rho_{3,7} - \frac{\rho_{3,7}}{C_6} \qquad (49)$$

$$\rho_{6,8} = \rho_{3,8} - \frac{\rho_{3,8}}{C_4} \qquad (50)$$

$$\rho_{6,10} = \frac{1}{C_6} \qquad (51)$$

Number of partners among clients and stabe partners

$$C_k = \frac{\sum_{k'}\left(C_{k'}\rho_{k',k}\sum_s X_{k'}^s\right)}{\sum_s X_k^s} \qquad (52)$$

Mixing matrix among clients and stable partners

$$\rho_{k,k'} = \frac{C_{k'}\rho_{k,k'}\sum_s X_{k'}^s}{C_k\sum_s X_k^s} \qquad (53)$$

$\rho_{k,k'}$ defines the proportion of total partnerships in group k that are formed with group k'. C_k is the total number of partnerships among clients and stable partners.

2. Model fitting: parameter values and sources

The epidemic was simulated using 10,000 different parameter sets obtained with Latin Hybercube Sampling. Parameters describing the natural history of infection and data informing the demography of the population (size and growth) are given in tables 1 and 2. The parameters allowed to vary were those describing sexual behavior as well as a couple describing the natural history of infection and they are described in table 3. Prior information on HIV prevalence among all TW-SW at different time points given in table 4 was used to select plausible epidemic fits. A run was accepted if it fitted within the low and high bounds of these prior limits. 498 runs were selected. The log likelihood of each selected epidemic fit was calculated using all available estimates of HIV prevalence among TW-SW, given in table 5. The 50 best epidemic fits based on the log likelihood were used to give the minimum and maximum values of intervention impact for each combination of interventions. This allows illustrating the range of most plausible intervention impact based on epidemic trajectories consistent with known data.

Parameters	Description	Symbol	Prior Value mode [min-max]	Reference
Average transmission rate of HIV per sex act	if latent HIV infection	β^{3-4}	0·001 [0·0008-0·002]	[7,8]
Start of the epidemic in Lima	year		1975	[9,10]
Relative transmission rate per receptive anal sex act	ref. insertive anal sex	ξ	10[5-15]	[8,11]
Relative infectiousness in acute phase infection	ref. latent infection	RR^{2}	27	[4]
Relative infectiousness in pre-AIDS phase infection	ref. latent infection	RR^{5}	7·2	[4]
Relative infectiousness in AIDS phase infection	ref. latent infection	RR^{6}	0	[4]
Relative infectiousness of virally-suppressed individuals on ART	ref. latent infection	RR^{7-8}	0·25	[4,12]
Mean duration of acute phase infection	months	$1/\sigma_2$	3	[4,12]
Mean duration early latent phase CD4 >500	years	$1/\sigma_3$	1.19	[5]
Mean duration latent phase CD4 500-350	years	$1/\sigma_4$	3.03	[5]
Mean duration of late latent phase CD4 350-200	years	$1/\sigma_5$	3.85	[4,12]
Mean interval with elevated viral load, pre-AIDS	years	$1/\sigma_6$	1	[4,12]
Mean interval with AIDS before death	months	$1/\sigma_7$	10	[4,12]
Mean mortality rate on ART late start (CD4 <200)	$years^{-1}$	ι_8	0.015	Average from [13]
Mean mortality rate on ART late start (CD4 200-350)	$years^{-1}$	ι_9	0.003	[13]
Mean mortality rate on ART late start (CD4 350-500)	$years^{-1}$	ι_{10}	0.002	[13]
Mean mortality rate on ART late start (CD4 >500)	$years^{-1}$	ι_{11}	0.001	Assumption inferred from [13]
Proportion diagnosed linked to ART (CD4 <200)	proportion	ϑ_8	0.95	Peruvian National Institute of Heath
Proportion diagnosed linked to ART (CD4 200-350)	proportion	ϑ_9	0.90/0.95	idem
Proportion diagnosed linked to ART (CD4 350-500)	proportion	ϑ_{10}	0.80/0.95	idem
Proportion diagnosed linked to ART (CD4 >500)	proportion	ϑ_{11}	0.80/0.95	Assumption
Rate of entry to ART (CD4 <200)	$years^{-1}$	δ_8	2*	[6,14-16]
Rate of entry to ART (CD4 200-350)	$years^{-1}$	δ_9	0.07**	Fit to coverage
Rate of entry to ART (CD4 350-500) (*basic/enhanced scenarios, among TW-SW* only)	$years^{-1}$	δ_{10}	0.5/0.75	Assumption intervention
Rate of entry to ART (CD4 >500) (basic/*enhanced scenario, among TW-SW* only)	$years^{-1}$	δ_{11}	0.5/0.75	Assumption intervention
ART dropout rate (CD4 <200)	$years^{-1}$	ϖ_8	0.005	Peruvian National Institute of Heath
ART dropout rate (CD4 200-350) (baseline+ basic/enhanced scenarios)	$years^{-1}$	ϖ_9	0.010/0.005	idem
ART dropout rate (CD4 350-500) (baseline+ basic/enhanced scenarios)	$years^{-1}$	ϖ_{10}	0.015/0.005	idem
ART dropout rate (CD4 >500) (baseline+ basic/enhanced scenarios)	$years^{-1}$	ϖ_{11}	0.015/0.005	Assumption
Efficacy of condoms	proportion	Ψ	0.7	[17,18]

Table 1. Parameters describing the natural history of infection.*Achieved after 3 years and then linearly increasing to a rate of 6/year, corresponding to a 2 month wait before entering treatment at very low CD4; **achieved after 5 years and changes to 0.5/0.75 for TW-SW only in *basic* and *enhanced* intervention scenarios respectively.

Parameters	Description	Value	Reference
Population 15 to 49y	2007	4,767,148	[19]
Population 15 to 49y	1981	2,503,140	[20]
First report of AIDS cases in Peru		1983	[9]
Proportion of the population that are men in Lima, Peru	2007	0·489	[19]
Proportion of male-to-male sex in general male population		0·06	[21-23]
Proportion of the MSM/Trans population that are TW		0.05	[24]
Alternative estimate of the TW population size in Lima		20,000	[25]

Table 2. Demographic data in Lima used to determine TW-SW population size and start year of the HIV epidemic

Parameter	Description	Symbol	Distribution	min	max	peak	References
**Transmission probability UIAI**	/sex act	β	Triangle	0.0008	0.002	0.001	[8]
**Rate of exit sex work**	/year	$\tau_{k=1-6}$	Uniform	0.065	0.2	-	Allowed wide variation
**Rate of exit sex business for clients**	/year	$\tau_{k=7-9}$	Uniform	0.065	0.2	-	Allowed wide variation
**Rate of exit for stable partners**	/year	$\tau_{k=10-12}$	Uniform	0.065	0.2	-	Assumed to be equal to TW-SW
**Population size at start**	(in 1975)	N_0	Uniform	30000	50000	-	Based on demographic info (T2)
**Proportion of the population that are TW-SW**		κ*	Uniform	0.1	0.2	-	Based on estimate of TW pop size (T2)
**Proportion TW-SW excl. receptive**		κ*	Triangle	0.7	0.9	0.8	[26]*
**Proportion of other TW-SW excl. insertive**		κ*	Triangle	0.4	0.6	0.5	[26]*
**Proportion TW-SW who have a stable partner**		κ*	Triangle	0.2	0.4	0.3	[26]*
**Number of partners**	/year	C_k	Triangle	50	300	250	[25]
**Number of sex acts in a SW partnership**	/partnership/year	$\omega_{k=1-6,k'=7-9}$	Uniform	1	10	-	Allows multiple contacts per client
**Number of sex acts in a stable partnership**	/partnership/year	$\omega_{k=1-6,k'=10-12}$	Triangle	25	100	50	Arbitrary based on reports from heterosexual
**Condom use with clients**	Proportion of sex acts protected at start	$\Phi_{k=1-6,k'=7-9}$	Triangle	0.24	0.54	0.34	[26]*
**Condom use with stable partners**	Proportion of sex acts protected at start	$\Phi_{k=1-6,k'=10-12}$	Triangle	0.15	0.35	0.25	[26]*
**Factor for linear condom increase**	Between 1985 and 1995		Uniform	1.5	3	-	[27] (rough inference)
**Relative risk receptive anal sex vs insertive**		ξ	Triangle	5	15	10	[8,11]
**Mean duration of stable partnership**	Years	ϖ	Triangle	0.4	3	1	Arbitrary based on [28] and discussions with local experts
**Constant multiplying the force of infection among stable partners of TWSW**			Uniform	1	4	-	Arbitrary based on report of high number of partners in [28]

Table 3. Parameters allowed to vary during fitting process
***subsample of those reporting paid sex, Silva-Santisteban, personal communication. The values obtained from this sub-sample were 0.75 and 0.5 condom use with clients and stable partners respectively, but we assumed this was much lower at the start of the epidemic and increased by a factor of 1.5 to 3 between 1985 and 1995, hence the parameter values used in the model were 0.34 and 0.25 respectively.**

Year of observation	Lower bound (%)	Upper bound (%)
**1980**	0	60*
**1990**	0	60*
1996	*17**	46
2002	*16**	38
2006	*16**	33*
2011	*16**	31*

Table 4. HIV prevalence bounds for the selection of epidemic fits
Legend: The bounds correspond to the confidence intervals reported for each surveillance round among MSM and TW in Lima. * The 1980 and 1990 higher bounds are an assumption based on experts' opinion. The lower bound for the 1996 and 2002 estimates were corrected down as other studies suggest lower prevalence in that period and during previous work with HIV experts in Lima involving a consultation with experts concerns were expressed about the high values obtained. These related to the sampling method and the fact that financial incentives were given to peer recruiters. These bound were corrected for the exclusion of HIV positive participants aware of their status as explained in table 5.

Year of observation	(Effective) Sample Size	HIV prevalence estimate (%)	Reference
**1996**	48	33.3	[29,30]
**1998**	134	34.3	[29]
**2000**	96	44.8	[29]
**2002**	255	32.2	[29]
**2006**	95	24.4*	[31]
**2008**	208	17.8	[32]
**2009**	459	29.6	[26]
**2011**	368	26.4*	[31]

Table 5. Prevalence data for Lima to calculate log likelihood of epidemic fits

Figure 3 presents HIV prevalence and ART coverage among TW-SW in the baseline scenario for the 50 simulations used in the analysis. The red curve corresponds to the best fit and the red crosses correspond to the data.

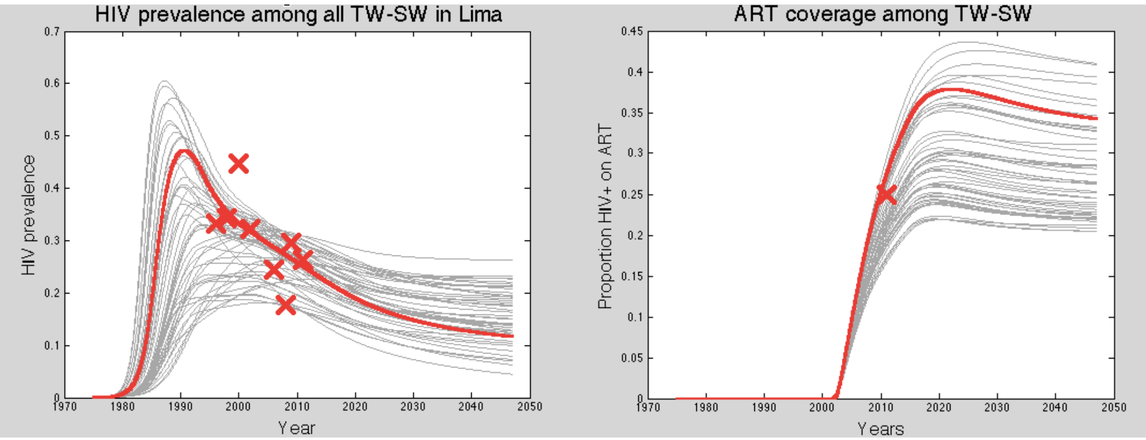

Figure 3. HIV prevalence (A) and ART coverage (B) among HIV positive TW-SW in Lima for the 50 best epidemic simulations

3. Costing

The costing of interventions was implemented as part of the health system capacity evaluation component of this study. Several sources were used to obtain local estimates of the costs of the interventions implemented in the model, including interviews with key informants, a review of grey literature and government reports in particular, and use of public databases. We contacted physicians and nurses at the Peruvian Ministry of Health (MINSA) to obtain information on the salary of the different healthcare providers involved in HIV care and the time each allocates to a routine ART monitoring patient visit. Healthcare providers and their time allocation in the context of PrEP were based on experience from the PrEP Ole study, with the assumption that it would be provided within the existing public HIV care system.
The cost of biomedical tests was based on the Peruvian National Institute of Health (INS) catalogue,[33] which is accessible to all for transparency.
The cost of ART drugs was based on a national study, which specifically calculated costs each region.[34] The costs of communication strategies were based on a costing effort carried out in the context of the Multisectoral Strategic Plan (PEM is the acronym in Spanish) of MINSA for the control of STIs and HIV/AIDS in Peru.[35] This was a participatory process originally motivated by the Millennium Development Goals, which resulted in the development of a national plan to respond to the epidemic from 2007 to 2011,[36] laying out objectives, actions to achieve them and their corresponding budget. A new plan was formulated for 2015-2019,[35] following a series of meetings with governmental (including representatives from the Ministries of Interior, Justice and Human Rights, Education, Employment, Women and Vulnerable populations, Foreign Affairs and Office of the Attorney General) and non-governmental (national and international) agencies, stakeholders including healthcare providers and affected population and academia. The budget was used to inform our costing exercise.
Finally, a grant proposal[37] to Round 10 of the Global Fund to increase access to HIV services among marginalised populations included detailed costing of outreach activities and in particular it emphasized the use of mobile units to provide prevention information to MSM and TW. Given that our treatment and PrEP interventions required steep increases in testing, we incorporated mobile units as a central component of these interventions and based our costing on this comprehensive analysis.

PrEP intervention

The PrEP intervention cost included the cost of efavirenz/emtricitabine (generic or Truvada® price, depending on the scenarios investigated), the cost of recruitment through mobile units (staffed with a peer recruiter and a nurse as described in the previous section), the cost of clinical monitoring, including HIV rapid tests, renal function tests and personnel (quarterly visits with a physician and a nurse and biannual visit with a psychologist and a social worker in addition to the administrative charge at each visit). It also included the cost of condoms, with the PrEP program assumed to cover 50% of protected sex acts among users. The cost of rapid and confirmatory tests among potential users diagnosed HIV positive at recruitment was also taken into account. For each person on PrEP, 1/(1-HIV prevalence) were tested and therefore 1/(1-HIV prevalence)-1 tested positive and required a confirmatory test. The cost of a yearly communication strategy to raise awareness of PrEP among TW-SW was also included. The total cost of one year of PrEP including recruitment, drugs and clinical monitoring was estimated at USD 105 with generic drugs and USD 1470 with Truvada.
The price of efavirenz/emtricitabine available through the PAHO Strategic Fund is of USD 5.25/month (30 tablets)[38], corresponding to USD 63/person/per year and we accounted for a 15% freight cost and a 4.25% indirect fee for buying through the fund, adding up to USD 75/person/year.
The additional cost per person associated with the distribution of condoms, additional rapid tests and confirmatory tests used on HIV positive potential users and communication strategy varied depending on the number of sex acts per person, the HIV prevalence and the total population of TW-SW but these were negligible. In addition, identifying undiagnosed HIV infections represents a desirable outcome for the HIV program, conditional on referral to treatment and care services. This synergy was not accounted for in the analyses.

PrEP	Proportion of time	Times per year	Cost	Reference
PrEP drugs (Generic/Truvada)	-	12	6.3/120	[38]/[39]
Recruitment with mobile units	-	1	12	See table 8
HIV rapid test	-	4	2.2	PEM
Renal function	-	2	1.6	INS
Physician (10 minutes)	0.2	4	9.4/hr	Communication from HIV physicians at MINSA
Nurse (10 minutes)	0.2	4	4.7/hr	
Psychologist (30 minutes)	0.5	1	4.7/hr	
Social worker (30 minutes)	0.5	1	4.7/hr	
Administrative charge	0.2	4	3.1/hr	
Condoms	50% of all protected sex acts		0.3	PEM
Additional recruitments with mobile units (tested positive)	1/(1-prevalence)-1 of the number receiving PrEP		12	See table 8
Additional rapid tests for HIV positives at recruitment			2.2	MINSA
Communication strategy	-	0.1	12500.0	PEM
Communication strategy material	-	1	2500.0	

Table 6. Components and corresponding cost estimates for a PrEP intervention

HIV treatment interventions

The HIV treatment intervention included the cost of ART drugs, the cost of clinical monitoring including consultations and biomedical tests (CD4 count, viral load and hematology tests) as well as the transport of samples. The cost of ART drugs was based on the cost of ARV drugs and the cost of monitoring and treatment initiation, The cost of ARV drugs was calculated as the total expenditure on ART medications reported by the Peruvian Ministry of Health (22.5 million soles, equivalent to 7 million USD) over the total number of patients on treatment covered by the Ministry of Health (35,000), corresponding to USD 200/person/year. Monitoring was estimated at USD 309/person/year; leading to a total of USD 509/person/year for treatment and monitoring. This is higher than estimated by Vargas[34], at USD 409/patient/year, including a USD 115/patient/year consultation cost, but our estimates accounted for transportation costs and a more detailed list of biomedical tests and personnel time required at each visit. The cost of ART initiation was estimated at USD 124/person and included the cost of additional bio-medical tests (Hepatitis B, Syphilis and Tuberculosis) as well as six additional monitoring appointments (with physician, nurse, pharmacist and including administrative charge) and one additional viral load test.

ART	Proportion of time	Times per year	Cost	Reference
ART drugs	-	1	200.0	Peruvian MoH
Physician (15 minutes)	0.25	4	9.4/hr	Communication from HIV physicians at MINSA
Nurse (15 minutes)	0.25	4	4.7/hr	
Psychologist (30 minutes)	0.5	2	4.7/hr	
Social assistant (30 minutes)	0.5	2	4.7/hr	
Pharmacist (5 minutes)	0.085	4	6.3/hr	
Administrative charge (10 minutes)	0.2	4	3.1/hr	
CD4 count	-	2	33.1	INS
Viral load	-	2	75.6	
Hematology tests	-	2	31.3	
Transport of samples	0.04	2	15.6	
ART initiation				
Testing through mobile unit			12	See table 8
HIV rapid test	Number new ART patients/prevalence		2.2	MINSA
Confirmatory HIV test	-	1	12.5	INS
Hepatitis B test	-	1	6.6	
Syphilis test	-	1	4.4	
Tuberculosis test	-	1	9.4	
Appointments	6 additional but only one extra viral load			Treatment guidelines
Testing campaign	-	0.1	12500.0	PEM
Testing campaign material	-	1	2500.0	

Table 7. Components and corresponding cost estimates for ART treatment interventions

Screening with mobile units

Both the treatment and the PrEP interventions required a drastic upscale in testing rates among the TW-SW population. Mobile units have proven effective in reaching this population in Lima and were included and costed as part of the 2015 Global Fund proposal "Expansion of the HIV national response in key and vulnerable populations in urban areas and Amazonian region of Peru".[37] Given it is a strategy that was adopted as part of the national plan to improve access to HIV services and it is currently being scaled up we considered it the most feasible and rapid solution to achieve the coverage required in our scenarios. Five components were considered in the costing of mobile units: transport and communication, human resources, staff training, population size estimation study and mapping study of the socialisation venues. Based on discussions with stakeholders and a proposal developed for the Global Fund, it was estimated that 10 mobile units would be required to cover Lima and Callao. The costs per mobile unit are disaggregated below. The transport and communication component included the mobile unit (and vehicle insurance, gas and maintenance) and a phone and tablet to communicate with health facilities and other mobile units. The mobile unit, phone and tablet were assumed to last for 5 years on average and therefore their cost was spread over this period. Each mobile unit was assumed to be staffed by a nurse, a counselor, two peer promoters and a driver. Their monthly salaries are provided in table 8 as well as the monthly cost of insurance against accidents. The cost of the training component, consisting of a yearly 3-day workshop and a yearly 3-day feedback and evaluation meeting was calculated based on the establishment of 10 mobile units. The costs are however expressed for a single mobile unit for consistency. The cost of the consultancy to develop the workshop and design the course book was assumed to cover 5 years of training and was therefore spread over that period. The cost of the evaluation meeting was assumed to be the same as that of the training workshop, excluding the consultancy and course book printing costs. The cost of a population size estimation study and a mapping of socialisation venues were included as these were considered key to an effective outreach of the mobile units. Both studies included a consultancy, a public presentation with experts and the production of a report and were assumed to be valid for a period of 5 years, with the costs spread over this period.

The total yearly operation cost of one mobile was estimated at USD 43, 128, corresponding to USD 3,594 per month. It was estimated that each unit would test 300 TW-SW per month based on 3 days of operation per week for 3 hours per day and 8 tests per hour. The cost of testing a TW-SW through the mobile unit was calculated at the total monthly cost of operation of the mobile unit, divided by 300 tests and including the cost of the rapid HIV test (USD 2.2), corresponding to USD 14.2 per test.

Screening			
	Times per year	Cost	Reference
Transport and communication			
Mobile unit	0.2	13125.0	GF proposal 2015
Vehicle insurance	1.0	1000.0	
Gas	1.0	4050.0	
Maintenance	1.0	187.5	
Mobile phone	0.2	31.3	
Mobile phone charges	1.0	337.5	
Tablet	0.2	93.8	
Snacks	1.0	450.0	
Total		8675.0	
Human resources			
Nurse	12.0	825.0	GF proposal 2015
Counsellor	12.0	825.0	
Two peer promoters	12.0	625.0	
Driver	12.0	468.8	
Insurance against accidents	12.0	16.3	
Total		33120.3	
Training			
Consultancy and design of course book	0.2	546.9	GF proposal 2015
Course book printing	0.2	54.7	
Course material	1.0	16.4	
Rent for 3 day workshop	1.0	58.6	
Rent of equipment	1.0	17.6	
Transport for participants	1.0	13.7	
2 training facilitators	1.0	97.7	
Communications	1.0	3.9	
Lunch and dinner for 3 days	1.0	123.0	
Total for training meeting		451.2	
Feedback and evaluation meeting	1.0	330.9	
Total		782.0	
Population size estimation			
Consultancy	0.2	976.6	GF proposal 2015
Public presentation with experts	0.2	39.1	
Report	0.2	68.4	
Total		216.8	
Mapping of socialisation venues			
Consultancy	0.2	1562.5	GF proposal 2015
Public presentation with experts	0.2	39.1	
Report	0.2	68.4	
Total		334.0	
TOTAL cost of mobile unit per year		43128.1	
Cost of mobile unit per month		3594.0	GF proposal 2015
Number of tests per mobile unit per month*		300.0	
Cost rapid HIV test		2.2	
Cost per HIV test with mobile unit		14.2	

*assuming it runs 3 times per week for 3 hours per day and tests 8 people per hour

Table 8. Components and corresponding cost estimates for outreach mobile units to test TW-SW

Condom promotion

The condom promotion interventions consisted of the combination of a condom inundation, a condom campaign and condom use workshops. The condom campaign included a market and behaviours' study to tailor messages and advertising methods to the TW-SW population, the campaign development and the material for its yearly implementation. Both the market study and the campaign development costs were assumed to be spread over 10 years. The condom workshops were assumed to reach all TW-SW through a "train the trainer" method in which 10% of TW-SW are trained every year and share their knowledge with other TW-SW. These workshops aim to enhance condom negotiation skills and practical skills to facilitate and eroticize the use of condoms. The workshop development cost was spread over the 10 years of the intervention. The condom inundation aimed to have branded condoms and lubricant highly accessible at strategic locations and venues based on the market and behaviours study. The condom interventions with clients and with stable partners were assumed to include the same components but implied separate processes, meaning that each of these activities and costs were incurred for the two interventions. The number of condoms distributed was calculated based on the total number of additional protected sex acts compared to baseline over the intervention time period accounting for condom loss.

Condom promotion interventions			
	Times per year	Cost	Reference
Condoms			
Branded condom and lubricant	-	1.25	PEM x 4
Condom loss	-	25%	Assumption
Condom campaign			
Market/behaviours study	0.1	13437.5	PEM
Development	0.1	12500.0	
Material	1	2500.0	
Condom workshops			
Development	0.1	4375.0	PEM
Training cost/per person	1/10 TW-SW	39.1	

Table 9. Components and corresponding cost estimates for condom use interventions

90-90-90 UNAIDS target scenario with associated improvements in other prevention interventions

In order to reach the 90-90-90 targets, a scale up of testing was required corresponding to the number of people needed to test to have 90% of HIV positive TW-SW aware of their status. This was calculated as the number of new ART patients/(0.9x0.9xprevalence), so that if 2000 extra patients entered treatment that year and prevalence was 20% then 2000/0.9x0.9x0.2=12345 would need to be tested of whom 222 would be HIV positive but would not access treatment and the remainder would be HIV negative. This assumes HIV positive and negative people are as likely to get tested which is to some extent a conservative assumption but appropriate in the absence of testing motivations and enablers among this population. The additional costs of ART initiation for those entering treatment, drugs and monitoring are not shown in the table but are as described in table 7. Several components were considered necessary to achieve the diagnosis, treatment and viral suppression goals. As described in the main text, the current information system does not allow linking patients being diagnosed at STI clinics to patients accessing care at treatment centers. More importantly, there is no system to enable linkage through communication between these different healthcare providers and it therefore falls on the patient to seek treatment. The latter is particularly challenging for marginalised populations such as TW-SW, who often avoid healthcare centers for fear of discrimination. To address this, the enhanced intervention scenario included the design of a training module to sensitize healthcare professionals to the gender and sexual diversity issues and to the precarious situation of TW-SW in particular. The cost of the training module design was spread over the 10 years of the intervention and the training implementation was assumed to occur yearly to account for the turnover of healthcare providers and to cement the knowledge among those who remain. In order to achieve viral suppression among 90% of those on treatment, support from peer educators was incorporated into the program, assuming each peer educator would cover 10 people on treatment. Given that ART shortages have been repeatedly reported in Lima and Callao, leading to temporary treatment interruptions among patients, the program included a system to strengthen the drug supply chain.

This scenario also assumed an improvement of the prevention interventions. A peer educator program was included in the PrEP intervention to increase adherence, and also assumed that each peer educator would cover 10 users. In regards to the condom interventions, this scenario assumed that twice the number of TW-SW and TW-SW-SP would receive the condom use training interventions to increase use with clients and stable partners, respectively.

Increased efficiency of interventions			
	Times per year	Cost	Reference
PrEP			
Peer educator for adherence support/person	1	150.0	PEM
ART			
Testing scale up	Number of new ART patients/(0.9x0.9xprevalence)		Table 7
Information system to improve linkage to care	0.1	337500.0	PEM
Design of diversity training for health care professionals to improve linkage and retention	0.1	40000.0	
Sensitization of health professionals through diversity training	1	15000.0	
Peer educator to improve adherence/person on ART	1	150.0	
Strengthening of drug supply chain to prevent shortages	1	23437.5	
Condom use interventions			
Doubling the coverage of the condom workshop	1/5 TW-SW	39.1	PEM

Table 10. Components and corresponding cost estimates for enhanced intervention scenario

4. Calculation of DALYs averted per HIV infection averted

DALYs averted per HIV infection averted among Peruvian TW-SW, clients and stable partners were calculated based on Fox-Rushby and Hanson using the 2010 Global burden of disease disability weights for HIV. DALYs correspond to the sum of years lost due to disability (YLD) and years of life lost as a result of early death (YLL). The level of disability varies depending on the disease stage and three stages are considered for HIV: pre-AIDS (corresponding to the period between infection and symptomatic AIDS, and therefore different from the pre-AIDS stage defined in the mathematical model, which corresponds to the high viral load period immediately preceding AIDS), AIDS and ART. The disability weights for HIV, AIDS and ART were estimated at 0.221, 0.547 and 0.053 respectively.[40]

DALYs per HIV infection averted= $YLD_{pre\text{-}AIDS} + YLD_{AIDS} + YLL$
Average DALYs per HIV infection averted weighted for ART= $YLD_{pre\text{-}AIDS}$ +ART coverage*($YLD_{ART} + YLL_{ART}$)+ (1-ART coverage)*($YLD_{AIDS} + YLL_{AIDS}$)

A number of assumptions are involved in the calculation of DALYs that are linked with value judgments and outlined below. An increased value was attributed to years of disability and life saved early after the (averted) infection compared to later in the progression of disease. This reflects a preference for the present or short-term future versus long-term future, but at the individual level, as opposed to at the population level as described above when discounting benefits and costs from the interventions. The standard 3% discount rate was used to account for this. The calculation of DALYs allows accounting for the increased value that may be attributed to life at certain ages than others (with young age valued more than infancy or old age, based on productivity). We decided not to use age weighting based on the fact that TW-SW work in the informal sector and need to do so throughout their lives, independently of old age. Little is known about clients and stable partners of TW-SW, but given this is a low-income environment, we assumed they would also work in the informal sector through old age. Although some studies use a standard life expectancy estimate (that of Japanese women is often used as it represents the highest level of health achieved) to calculate DALYs, for cost-effectiveness analyses it is important to use local estimates as policy decisions are made on the bases on contextual conditions. We therefore calculated DALYs averted per HIV infection averted for TW-SW assuming a life expectancy of 60 years, based on the high mortality rate among this population. For their clients and stable partners, we calculated DALYs averted per HIV infection averted assuming a life expectancy of 70 years, which is approximately 5 years lower than the standard life among Peruvian men, based on preliminary evidence suggesting high-risk behaviors among partners of TW-SW and low socio-economic status. An additional assumption when incorporating ART into these calculations is the years of life extension provided by ART. In a study that pooled data from several cohorts of people on ART treatment in Europe and North America[41], it was estimated that among all patients treated after 2008, ART increased life expectancy by approximately 35 years. A young man starting treatment at age 20 in Europe was expected to live until age 67, compared to a population life expectancy of 79. Mortality in the first year of ART is much higher and therefore life expectancy among those on ART with a CD4 count higher than 350cells/mm^3 were expected to have a life expectancy equivalent to that of an HIV negative person. Early start is a key condition for such a successful outcome and similar cohort studies in Latin America and the Caribbean[42] (including in Peru) suggest that mortality in the first year of treatment is much higher in this region compared to Europe, the U.S. and Canada (57.8/1000 person years versus 24.9/1000 person years). Later ART initiation and less effective treatment regimens are largely accountable for these high rates. Mortality after the first year was slightly lower in Latin America and the Caribbean (12.6/1000 person years versus 15/1000 person years), likely

reflecting higher losses to follow up. Overall mortality after 3 years on ART was 8.3% in Latin America and the Caribbean versus 5% in Europe and North America (the time periods are not exactly comparable as the former investigated the 2000-2014 period while the latter investigated the 1996-2013 period). These data suggest life expectancy on ART is lower in Peru compared to Europe and North America. In addition, this and other studies[43] highlighted the importance of structural and socio-economical barriers to access to HIV care as key determinants of life expectancy. Given the situation of marginalization among TW-SW and their sexual partners, their difficulties accessing health care and their challenges with adherence, we assumed a significantly lower extension of life expectancy on ART among this population (20 years). This is lower than the life expectancy on ART estimated in Uganda [44]among the general population (26 years) or in Rwanda[45]. We explored a second scenario assuming a lower extension of life expectancy on ART (10 years) based on a modelling study among female sex workers in India[46] and on a study in Peru showing very short life expectancy among patients in low resource settings (6.6 years).[47,48]
ART coverage at baseline was assumed to be 35% based on our best fit model projection.
One HIV infection averted translated to 11.6 and 14.5 DALYs averted among TW-SW and their sexual partners respectively after discounting and without including age weighting when assuming 20 years of life extension on ART and 13.6 and 16.1 DALYs averted when assuming 10 years of life extension on ART.[49]

		TW-SW		Clients and stable partners	
		Without ART	With ART	Without ART	With ART
Standard life expectancy		60		70	
Life expectancy with HIV/years of life extension on ART		9 years HIV, 1 year AIDS	20/10	9 years HIV, 1 year AIDS	20/10
Age weighting modulation factor	K	0.00	0.00	0.00	0.00
Constant	C	0.1658	0.1658	0.1658	0.1658
Discount rate	d	0.03	0.03	0.03	0.03
Age parameter	Beta	0.00	0.00	0.00	0.00
Years of life lived with disability (HIV)					
Age at onset of disability	a	24.00	24.00	24.00	24.00
Duration of disability	L	9.25	8.00	9.25	8.00
Disability weight	D	0.221	0.221	0.221	0.221
Years of life lived with disability (AIDS/ART)					
Age of onset of disability	a	33.25	32.00	33.25	32.00
Duration of disability	L	0.83	22.08/12.08	0.83	22.08/12.08
Disability weight	D	0.547	0.053	0.547	0.053
Years of life Lost					
Parameter from the age weighting function	b	0.00	0.00	0.00	0.00
Age of death	a	34.08	54.08/44.08	34.08	54.08/44.08
Potential years of life left at time of death	L	25.92	5.92/15.92	35.92	15.92/25.92
YLD calculation_HIV		1.79	1.57	1.79	1.57
YLD_AIDS		0.45	0.86/0.54	0.45	0.86/0.54
YLL calculation		18.02	5.42/12.66	21.99/16.25	12.66/18.02
YLD_AIDS and YLL		20.25	7.85/14.77	24.22/18.37	15.09/20.13
Discounted					
YLD calculation_HIV		1.79	1.57	1.79	1.57
YLD_AIDS		0.34	0.67/0.42	0.34	0.67/0.42
YLL calculation		13.31	2.20/6.93	16.25	5.13/9.86
YLD_AIDS and YLL		15.44	4.44/8.92	18.37	7.38/11.86
ART coverage		0.35		0.35	
DALY/infection		15.91/18.33		21.02/22.79	
DALY/infection discounted		11.59/13.16		14.53/16.09	

Table 11. Detailed calculation of DALYs averted per HIV infection averted among TW-SW and their clients and stable partners in Lima, Peru. YLLs: years of life lost; YLDs: years of life lived with disability.

5. Cost effectiveness

The impact of each intervention in isolation and all the possible permutations of combinations of interventions were assessed over a 10 year period between 2016 and 2026 by estimating the number of infections averted as compared to the baseline scenario (i.e. subtracting the number of new infections happening in the intervention scenario over the 10 year period to the number of new infections happening in the baseline scenario over the same period). Cost-effectiveness was estimated by calculating the cost per DALY averted for each of the intervention scenarios. This allows making comparisons across countries and diseases and therefore provides a more standardised estimate of cost-effectiveness than presenting cost per infection averted. Costs and benefits were discounted at 3% to take into account the increased value attributed to the present as compared to the longer-term future. The cost-effectiveness thresholds (CET) considered were the low and high bounds of the CET estimated by Ochalek et al,[50] which consider demographic, epidemiologic and health expenditure variables for Peru (208 and 1300 USD/DALY averted respectively), which are significantly lower than the WHO CHOICE[51] CET of 1 GDP per capita/DALY averted corresponding to 6,045 USD/DALY averted, and close to the World Bank CET[52] adjusted to their inflated 2016 equivalent corresponding to 986 and 197 USD/DALY averted for a cost-effective and highly cost-effective intervention respectively.
The averted cost of ART treatment achieved through averting new infections occurring after the 10-year intervention period was not accounted here given the uncertainty in these costs in the long term. In addition, the use of DALYs averted per infection averted as implemented in this study assumes that the infections that were averted over this 10 year period are averted in the long term, which might lead to an over-estimation of the benefits achieved through the interventions. However, averting infections over that 10-year period also results in averting secondary infections in the long term, which is not accounted for in the cost effectiveness analysis, potentially resulting in an under-estimation of the benefits achieved through the interventions. Lastly, while the synergistic effect of interventions on the epidemic was modeled, the synergy in terms of costs was not represented. In particular, both the PrEP and the treatment interventions required a scale up in testing efforts through mobile units, which would in practice be used concurrently to both diagnose new infections and recruit PrEP users and therefore the cost of testing under the PrEP intervention could be eliminated. We opted for a conservative approach in this analysis to consider instances in which the PrEP and testing interventions would occur independently of each other based on outreach strategies and user preferences.

6. Stakeholder analysis and health system capacity assessment

Stakeholder analysis

The aim of the stakeholder analysis **was to identify current views about an HIV combination prevention program for MSM/transwomen incorporating old (behavioral change, STI control) and new (improved testing linked to care, PrEP, early ARV for prevention – TasP) technologies as well as structural interventions (e.g. cost reduction, addressing stigma and discrimination, increasing access):**

- Describe perceptions, meanings and knowledge about PrEP and TasP, including beliefs in effectiveness, need for combination with condoms or lack thereof, concerns, and practical implications. Identify sub-groups within those populations more willing to use PrEP/TasP (e.g. younger people, people who dislike condoms, seronegative partners) and describe potential profiles of users.
- Describe perceptions of 'older' technologies (e.g. HTC, behavioral change communication, condom distribution, STI management) and the degree to which they have been 'updated' (e.g. couples HTC, content on treatment and discussion of seroadaptive practices in counseling) or maintain acceptable quality; identify perceptions about room for improvement.
- Describe perceptions about present levels of access (and barriers) to existing prevention tools, and potential structural changes needed to implement new prevention strategies such as PrEP and TasP.
- Describe perceptions about structural changes (e.g. cost reduction, addressing discrimination, increasing access within a human rights framework) needed to strengthen HIV prevention in general.

In the context of the modelling study, the stakeholder analysis provided data on acceptability, feasibility, appropriate coverage targets and scale-up times of both novel and (improved) existing interventions. To identify interventions to be considered in the combination prevention package the following interviews were carried out: 16 in-depth interviews and 4 focus groups with MSM and TW, semi-structured interviews with 7 health professionals, 4 decision makers and 5 community leaders between June and October 2014 in Metropolitan Lima; additional interviews and focus groups were conducted in Trujillo and Iquitos, respectively, in February-May 2015. Their views on available and novel prevention methods were gathered using the WHO guidelines for HIV in key populations as a reference. Realistic estimates of the coverage and time to scale-up of the interventions considered were obtained during a national HIV prevention consultation in November 2014 which gathered about 100 stakeholders representative of all parties concerned. Participants were split into three workgroups to discuss different aspects of combination prevention programmatic planning. One of the groups focused on the following modeling questions to inform this modeling study.

Workgroup III: Mathematical modelling of HIV combination prevention among MSM/TW in Peru			
13th of November 2014			
**Group**			
**Facilitador**			
**Key questions**	**Issues to consider**	**Conlusions**	
What interventiosn would be needed to achieve these goals?	Increase in testing coverage		
	Increase in treatment coverage		
	Increase in condom use		
	Early ART treatment		
	PrEP in selected populations		
	Other		
What would be realistic goals for the coverage increase of these services and for the time required to achieve it?	Increase in testing coverage		
	Increase in treatment coverage		
	Increase in condom use		
	Early ART treatment		
	PrEP in selected populations		
	Other		

Health system capacity assessment

The aim of the health system capacity assessment was **to identify capacity gaps in the health system to offer new HIV prevention technologies for MSM/TW, and costs of scaling up new technologies.**

The health system study assessed capacity, costs and needs highlighting gaps and barriers to the implementation of prevention and treatment services through (a) visits to facilities and completion of a structured observation guide, in dialogue with personnel in the facility; (b) collection of cost figures for all relevant expenditures (source: Results-based Budget, National Multisectoral Plan), key informant interviews, and review of costs with a larger number of stakeholders; (c) collection of relevant estimates of diverse indicators, including cascade coverage estimates from the Peruvian National Institute of Health. Prevention and treatment services were selected in Lima Metropolitana (i.e. Callao, Downtown Lima and Lima-South), in two coastal cities (Trujillo, Ica) and two cities in Amazonia (Iquitos, Pucallpa). This information was used in the modelling study to inform the interventions' design and cost.

7. Cost and incremental cost-effectiveness of interventions when assuming the branded (Truvada®) efavirenz/emtricitabine of USD 120/person/month

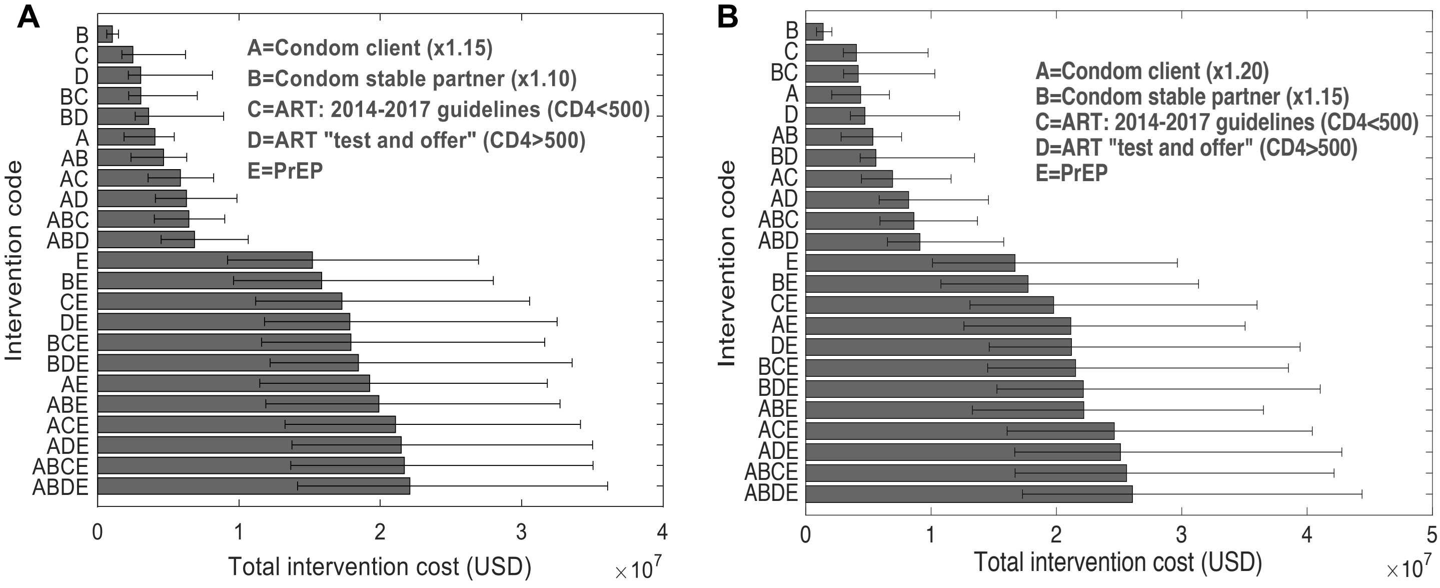

Figure 4. Total cost over 10 years for each intervention in the A) "*basic scenario*" and B) "*enhanced scenario*" assuming branded PrEP cost

Five intervention strategies were non-dominated for the basic scenario (Figure 5A): A, AC, AD, ABD and ABDE with an ICER of 125, 179, 202, 245 and 5884 USD/DALY averted, respectively. The ICERs for the A, AC, AD, ABD strategies were under the high bound CET (USD 1300/DALY averted) while strategies A, AC and AD were under the low bound CET (USD 208/DALY averted). The strategy combining all interventions including PrEP at current branded Truvada® price was not cost-effective under the specified CET.

Four intervention strategies were non-dominated for the enhanced scenario (Figure 5B): BC, AC, ABD and ABDE with an ICERs of 104, 155, 414 and 6047 USD/DALY averted, respectively.
BC and AC were cost effective under the low bound CET and ABD was cost effective under the high bound CET. The strategy combining all interventions including PrEP at current branded Truvada® price was not cost-effective under the specified CET.

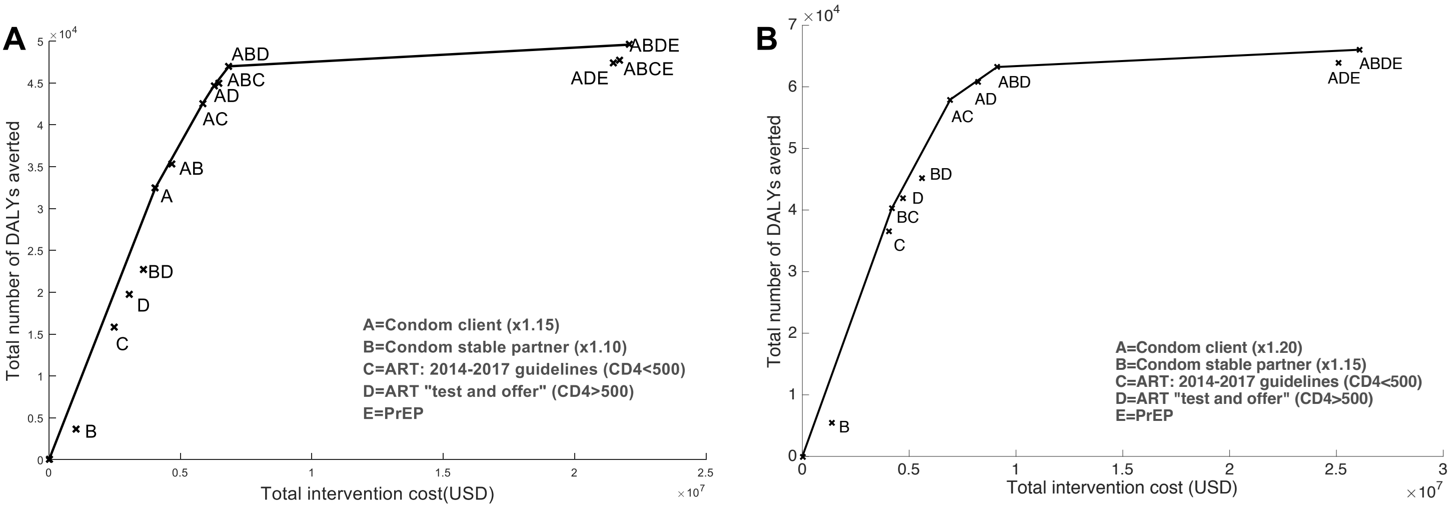

Figure 5. Incremental cost effectiveness graph showing the cost and number of DALYs averted for non-strongly dominated interventions in the A) "*basic scenario*" and B) "*enhanced scenario*" assuming branded PrEP cost

References

1. Poteat T, Wirtz AL, Radix A, et al. HIV risk and preventive interventions in transgender women sex workers. *Lancet* 2015; **385**(9964): 274-86.
2. Smith DK, Herbst JH, Zhang XJ, Rose CE. Condom Effectiveness for HIV Prevention by Consistency of Use Among Men Who Have Sex With Men in the United States. *Jaids-J Acq Imm Def* 2015; **68**(3): 337-44.
3. Silva-Santisteban A, Raymond HF, Salazar X, et al. Understanding the HIV/AIDS epidemic in transgender women of Lima, Peru: results from a sero-epidemiologic study using respondent driven sampling. *AIDS and behavior* 2012; **16**(4): 872-81.
4. Hollingsworth TD, Anderson RM, Fraser C. HIV-1 transmission, by stage of infection. *J Infect Dis* 2008; **198**(5): 687-93.
5. Lodi S, Phillips A, Touloumi G, et al. Time From Human Immunodeficiency Virus Seroconversion to Reaching CD4+ Cell Count Thresholds < 200, < 350, and < 500 Cells/mm(3): Assessment of Need Following Changes in Treatment Guidelines. *Clinical Infectious Diseases* 2011; **53**(8): 817-25.
6. UNAIDS. Peru: Epidemiological Fact Sheet on HIV and AIDS: Core data on epidemiology and response 2008 update. http://apps.who.int/globalatlas/predefinedReports/EFS2008/full/EFS2008_PE.pdf (accessed.
7. Baggaley RF, Garnett GP, Ferguson NM. Modelling the impact of antiretroviral use in resource-poor settings. *PLoS medicine* 2006; **3**(4): e124.
8. Baggaley RF, White RG, Boily MC. HIV transmission risk through anal intercourse: systematic review, meta-analysis and implications for HIV prevention. *Int J Epidemiol* 2010; **39**(4): 1048-63.
9. Patrucco R. Síndrome de Inmunodeficiencia Adquirida en el Perú (Sida). Estudios Inmunológicos. *Diagnóstico* 1985; **16**(5): 122-35.
10. CDC. Pneumocystis pneumonia--Los Angeles. MMWR Morb Mortal Wkly Rep. 1981.
11. Vittinghoff E, Douglas J, Judson F, McKirnan D, MacQueen K, Buchbinder SP. Per-contact risk of human immunodeficiency virus transmission between male sexual partners. *American journal of epidemiology* 1999; **150**(3): 306-11.
12. Donnell D, Baeten JM, Kiarie J, et al. Heterosexual HIV-1 transmission after initiation of antiretroviral therapy: a prospective cohort analysis. *Lancet* 2010; **375**(9731): 2092-8.
13. Smit M, Smit C, Cremin I, Garnett GP, Hallett T, de Wolf F. Could better tolerated HIV drug regimens improve patient outcome? *AIDS (London, England)* 2012; **26**(15): 1953-9.
14. UNAIDS. Informe Nacional de UNGASS, 2009.
15. San Francisco Department of Public Health. Annual HIV/AIDS surveillance report. 2005-2012.
16. Samji H, Cescon A, Hogg RS, et al. Closing the gap: increases in life expectancy among treated HIV-positive individuals in the United States and Canada. *PloS one* 2013; **8**(12): e81355.
17. Pinkerton SD, Abramson PR. Effectiveness of condoms in preventing HIV transmission. *Social science & medicine (1982)* 1997; **44**(9): 1303-12.
18. Weller S, Davis K. Condom effectiveness in reducing heterosexual HIV transmission. *Cochrane database of systematic reviews (Online)* 2002; (1): CD003255.
19. INEI. Censo de la poblacion. 2007. http://censos.inei.gob.pe/censos2007/.
20. INEI. Censo de la poblacion. 1981. http://www.inei.gob.pe/.
21. Caceres C, Konda K, Pecheny M, Chatterjee A, Lyerla R. Estimating the number of men who have sex with men in low and middle income countries. *Sexually transmitted infections* 2006; **82 Suppl 3**: iii3-9.
22. Garcia PJ, Holmes KK, Carcamo CP, et al. Prevention of sexually transmitted infections in urban communities (Peru PREVEN): a multicomponent community-randomised controlled trial. *Lancet* 2012; **379**(9821): 1120-8.
23. PAHO. Modos de Transmisión del VIH en América Latina: Resultados de la aplicación del modelo. Lima: MINSA, 2009.
24. Caceres CF, Konda K, Segura ER, Lyerla R. Epidemiology of male same-sex behaviour and associated sexual health indicators in low- and middle-income countries: 2003-2007 estimates. *Sexually transmitted infections* 2008; **84 Suppl 1**: i49-i56.
25. Caceres S, Santisteban et al. Estudio sobre los factores que incrementan la vulnerabilidad al VIH, riesgos de la femenización corporal, necesidades de educación y laborales de la población Trans en las regiones intervenidas. 2012.
26. Silva-Santisteban A, Raymond HF, Salazar X, et al. Understanding the HIV/AIDS Epidemic in Transgender Women of Lima, Peru: Results from a Sero-Epidemiologic Study Using Respondent Driven Sampling. *AIDS Behav* 2011.
27. MINSA. Base de Datos y Referencias para una Estrategia Basada en Evidencia. Fortalecimiento del Plan Estratégico Multisectorial para la Prevención y Control de las ITS, VIH y SIDA en el PERÚ:2007-2011. 2012.
28. Operario Dpc. Data from a subsample of TGW that reported paid sex who participatedin a study of TGW and their main partner in the San Francisco Bay area. This is unpublished but part of the Operario and Nemoto (2011) study. 2013.

29. Sanchez J, Lama JR, Kusunoki L, et al. HIV-1, sexually transmitted infections, and sexual behavior trends among men who have sex with men in Lima, Peru. *Journal of acquired immune deficiency syndromes (1999)* 2007; **44**(5): 578-85.
30. Tabet S, Sanchez J, Lama J, et al. HIV, syphilis and heterosexual bridging among Peruvian men who have sex with men. *AIDS (London, England)* 2002; **16**(9): 1271-7.
31. Sánchez P, Lama. Estudio de Vigilancia Epidemiológica de ITS y VIH en Hombres que Tienen Sexo con Hombres Comparando las Metodologías de Reclutamiento: Muestreo por Conveniencia, Muestreo por Tiempo y Espacio y el Muestreo Dirigido por Participantes. 2011.
32. communication) Cp. CPOS study among TGW in Lima, baseline.
33. Instituto Nacional de Salud. Catalogo de precios y/o productos. 2017. http://www.portal.ins.gob.pe/en/10-servicios-web-para-ciudadanos/556-catalogo-de-precios-de-productos-y-o-servicios (accessed September 2015).
34. Vargas V. The new HIV/AIDS program in Peru: the role of prioritizing and budgeting for results2015. http://documents.worldbank.org/curated/en/167721468284339929/pdf/942600WP00PUBL0IV0AIDS0Program0Peru.pdf (accessed.
35. Ministerio de salud. Plan estrategico multisectorial de prevencion y control de las ITS/VIH y SIDA, 2015-2019 Lima, Peru; 2015.
36. Ministerio de Salud del Perú Plan Estratégico Multisectorial VIH-SIDA 2007 - 2011. 2006.
37. Global Fund. Expansion of the HIV National Response in key and vulnerable populations in urban areas and Amazonian region of Peru. 2015. https://www.theglobalfund.org/en/portfolio/country/grant/?k=16142633-d2a8-40fb-9508-8bc9fb834f96&grant=PER-H-PATH (accessed July 2017).
38. Fund PAHOPS. Long term agreement: antiretroviral medicines. 2018. https://www.paho.org/hq/index.php?option=com_docman&view=download&category_slug=product-list-references-prices-8778&alias=32472-product-prices-antiretroviral-until-dec-31-2018-472&Itemid=270&lang=en (accessed September 12 2018).
39. Observatorio de Productos Farmaceuticos (Sistema National de Informacion de Precios). Peruvian Ministry of Health (MINSA). TRUVADA 200 mp+300 mg Tableta - Capsula. 2017. http://observatorio.digemid.minsa.gob.pe/Precios/ProcesoL/Consulta/BusquedaGral.aspx?grupo=5321*3&total=2*1&con=200*mg*$*300*mg&ffs=3&ubigeo=15&cad=TRUVADA*200*mg*$*300*mg*Tableta*-*Capsula (accessed July 2017.
40. Salomon JA, Vos T, Hogan DR, et al. Common values in assessing health outcomes from disease and injury: disability weights measurement study for the Global Burden of Disease Study 2010. *Lancet* 2012; **380**(9859): 2129-43.
41. Antiretroviral Therapy Cohort C. Survival of HIV-positive patients starting antiretroviral therapy between 1996 and 2013: a collaborative analysis of cohort studies. *The lancet HIV* 2017.
42. Carriquiry G, Fink V, Koethe JR, et al. Mortality and loss to follow-up among HIV-infected persons on long-term antiretroviral therapy in Latin America and the Caribbean. *J Int Aids Soc* 2015; **18**: 20016.
43. Katz IT, Maughan-Brown B. Improved life expectancy of people living with HIV: who is left behind? *The lancet HIV* 2017.
44. Mills EJ, Bakanda C, Birungi J, et al. Life expectancy of persons receiving combination antiretroviral therapy in low-income countries: a cohort analysis from Uganda. *Annals of internal medicine* 2011; **155**(4): 209-16.
45. Egger M, Johnson LF. Estimating trends in life expectancy in HIV-positive individuals. *Lancet Glob Health* 2015; **3**(3): e122-3.
46. Vassall A, Pickles M, Chandrashekar S, et al. Cost-effectiveness of HIV prevention for high-risk groups at scale: an economic evaluation of the Avahan programme in south India. *Lancet Glob Health* 2014; **2**(9): e531-40.
47. Munoz M, Bayona J, Sanchez E, et al. Matching social support to individual needs: a community-based intervention to improve HIV treatment adherence in a resource-poor setting. *AIDS and behavior* 2011; **15**(7): 1454-64.
48. Bern C, Kawai V, Vargas D, et al. The epidemiology of intestinal microsporidiosis in patients with HIV/AIDS in Lima, Peru. *The Journal of infectious diseases* 2005; **191**(10): 1658-64.
49. Fox-Rushby JA, Hanson K. Calculating and presenting disability adjusted life years (DALYs) in cost-effectiveness analysis. *Health Policy Plan* 2001; **16**(3): 326-31.
50. Ochalek J, Lomas J, Claxton K. Cost Per DALY Averted Thresholds for Low- and Middle-Income Countries: Evidence From Cross Country Data. *CHE research paper 122*, 2015. (accessed February 2018).
51. WHO. Cost effectiveness and strategic planning (WHO-CHOICE). 2015. http://www.who.int/choice/costs/CER_thresholds/en/ (accessed October 2015 2015).
52. World Bank. World development report. Washington, DC; 1993.
